# Progressive intestinal tumor cell plasticity, Myc activation, and loss of Lgr5^+^ tumor stem cell lineage commitment upon Wnt depletion

**DOI:** 10.1126/sciadv.aeb8564

**Published:** 2026-07-10

**Authors:** Marika Lassila, Fatemeh Seyednasrollah, Cinzia Bessone, Maritta Räisänen, Inkeri Vuori, Davide G. Berta, Johanna Aspholm, Tatiana V. Petrova, Lauri A. Aaltonen, Kari Alitalo, Pauliina Kallio

**Affiliations:** ^1^ Translational Cancer Medicine Program, Research Programs Unit, University of Helsinki, Helsinki 00014, Finland.; ^2^ Medicum/Department of Medical and Clinical Genetics, University of Helsinki, Helsinki 00014, Finland.; ^3^ Applied Tumor Genomics Research Program, Research Programs Unit, University of Helsinki, Helsinki 00014, Finland.; ^4^ Department of Fundamental Oncology, University of Lausanne, and Ludwig Institute for Cancer Research Lausanne, Lausanne, Switzerland.; ^5^ Swiss Institute for Experimental Cancer Research (ISREC), School of Life Sciences, EPFL, Lausanne, Switzerland.; ^6^ Department of Pathology, HUS Diagnostic Center, Helsinki University Hospital, Helsinki 00029, Finland.; ^7^ Wihuri Research Institute, Biomedicum Helsinki, University of Helsinki, Helsinki FI-00014, Finland.

## Abstract

Plasticity, the capability of tumor cells to go through phenotypic transitions, promotes colorectal cancer (CRC) progression and treatment resistance. Although plasticity is evident in advanced CRCs, little is known about plasticity in early-stage tumors and tumor stem cells. Here, we demonstrate that a plastic cell state (PCS) is present already in polyps from patients with familial adenomatous polyposis and in mouse intestinal adenomas, in which PCS is associated with *PROX1*^+^ tumor stem cells. We furthermore analyzed progressive plasticity upon loss of the canonical wingless-related integration site (Wnt) effector *Tcf7* or *Lef1* in *Apc* mutant mice. Deletion of either gene led to emergence of new plastic tumor cell populations, failure of leucine-rich repeat–containing G protein-coupled receptor 5 (Lgr5) tumor stem cell differentiation into enterocyte-like cells, enhanced Myc pathway activation, and increased tumor cell proliferation and tumorigenesis. Together, we demonstrate that PCS is associated with early CRC development and identify multiple potentially druggable mechanisms activated during progressive tumor cell plasticity.

## Teaser

Tumor cell plasticity emerges in intestinal polyps and adenomas and drives the activity of potentially druggable pathways.

## INTRODUCTION

Colorectal cancer (CRC) is one of the leading causes of cancer-related mortality worldwide (*1*). The prognosis of CRC is greatly affected by the presence of distant metastasis, and recent studies have shown, that among other factors, tumor cell plasticity drives CRC metastasis (*2*–*5*). Phenotypic plasticity, which is one of the cancer hallmarks, includes intralineage and translineage plasticity (*6*, *7*). Intralineage plasticity allows cells to revert from mature states back to progenitor states and promotes inhibition of differentiation (*6*, *7*). Upon translineage plasticity, cells can transdifferentiate from their own developmental lineage toward distinct cell lineages, as has been reported in CRC (*6*–*10*). In the context of CRC, translineage plasticity is characterized by loss of the canonical intestinal cell identity and the emergence of oncofetal and noncanonical transcriptional gene programs (*8*–*10*). It has been suggested that one of the initiating steps of progressive plasticity in CRC is loss of wingless-related integration site (Wnt)/β-catenin signaling (*9*). However, the steps following the loss of canonical tumor cell state via depletion of canonical Wnt signaling are incompletely characterized.

The Wnt/β-catenin signaling pathway is hyperactivated in most patients with CRC due to mutations in the *Adenomatous polyposis coli* (*APC*) gene, which is considered to initiate the adenoma-carcinoma sequence of CRC development (*11*–*14*). One of the target genes of the Wnt/β-catenin signaling, leucine-rich repeat–containing G protein-coupled receptor 5 (LGR5), is expressed by CRC stem cells (*15*–*17*). Paradoxically, cells expressing low levels of Lgr5 can form CRC metastases (*18*). Moreover, pharmacological inhibition of the RAS-Gly12Asp (RAS-G12D) oncoprotein converted plastic *Emp1*^+^ metastatic cells to a WNT-driven *Lgr5*^+^ state (*19*). On the other hand, the Lgr5^+^ cancer stem cells are essential for liver metastasis in preclinical mouse models (*20*). Thus, the role of Lgr5^+^ tumor cells upon plasticity and metastasis remains complex. Another Wnt/β-catenin signaling target gene in CRC is transcription factor Prospero homeobox 1 (*PROX1*), which was previously shown to be expressed by treatment-resistant tumor stem cells (*21*–*23*). The *Lgr5*^+^ and *Prox1*^+^ tumor stem cells show distinct lineage commitment in *Apc^Min/+^* tumors (*23*).

To date, tumor cell plasticity has been studied in samples derived from patients with advanced CRC and in preclinical mouse tumor models harboring multiple mutations typical of advanced CRC (*8*–*10*, *19*, *24*). To address the early developmental steps of plasticity, we first investigated tumor cells in preclinical mouse tumor models containing only *Apc* deletion. Furthermore, we investigated polyp samples from patients with familial adenomatous polyposis (FAP), which harbor mutations in the *APC* gene. In both models, we found a plastic cell state (PCS) in the *PROX1*^+^ tumor–specific stem cells of polyps and adenomas, indicating the presence of plasticity already from the earliest phase of the adenoma-carcinoma sequence. In addition, we investigated whether deletion of Wnt/β-catenin signaling mediators, T cell factor *Tcf7* or lymphoid enhancer–binding factor *Lef1* (*11*), induces progressive plasticity as speculated in a previous study (*9*). To focus on the tumor stem cells, these deletions were induced using Lgr5-Cre^ERT2^ recombinase. Deletion of either *Tcf7* or *Lef1* led to a transcriptional shift, consistent with translineage plasticity, and emergence of additional highly plastic tumor cell populations. These deletions led also to increased intralineage plasticity, reflected in the failure of *Lgr5*^+^ stem cells to commit to enterocyte-like cells, and increased expression of Inhibitor of DNA binding 1-3 (*Id1-3*) and SRY-box transcription factor 9 (Sox9) that act as inhibitors of differentiation (*25*, *26*). Moreover, we found increased expression of transcripts involved in the noncanonical Wnt signaling, increased tumor cell proliferation, and accelerated tumorigenesis via enhanced Myc signaling, all of which represent previously undescribed features of progressive plasticity.

## RESULTS

### Tumor stem cells in intestinal human polyps and mouse adenomas are highly plastic

**Fig. 1.**
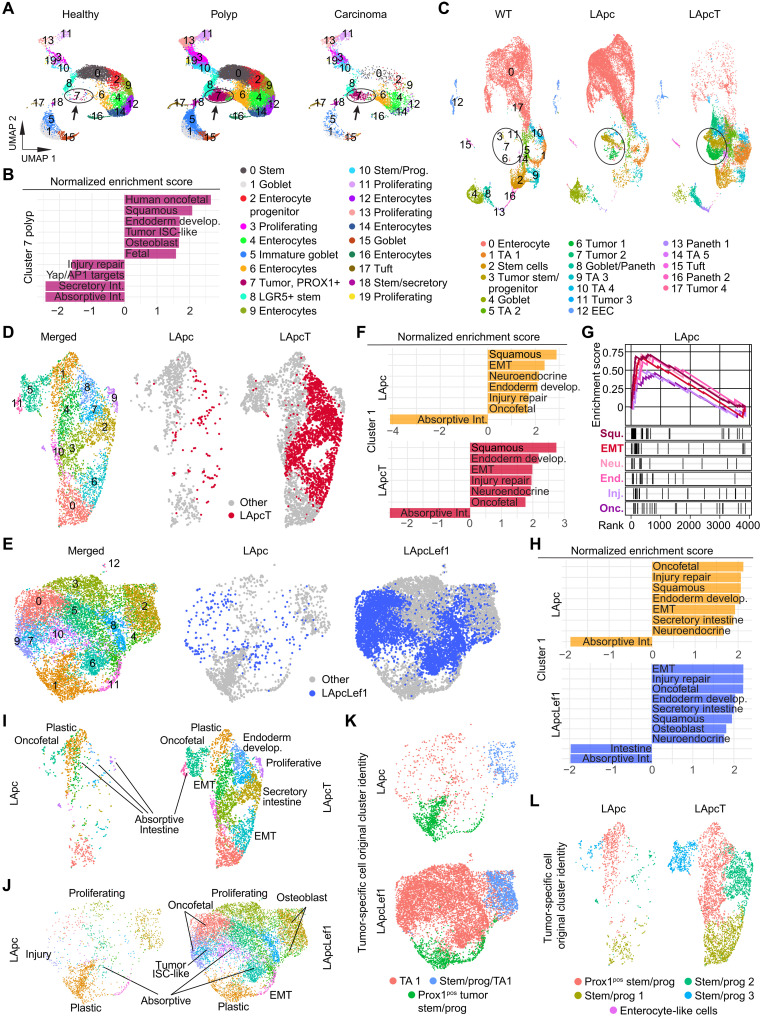
Plastic tumor stem cells are present in *APC* mutated large intestinal tumors. (**A**) Uniform manifold approximation and projection (UMAP) visualization of the *EPCAM*^+^ cells from large intestinal human healthy, FAP-polyp, and carcinoma samples. The tumor-specific cluster is encircled. (**B**) Normalized enrichment scores of canonical, noncanonical, and oncofetal gene sets analyzed using gene set enrichment analysis (GSEA) and fgsea R package applied to the cluster 7 polyp cells. Presented are gene sets with adjusted *P* (padj.) < 0.05. develop., development. ISC, intestinal stem cell. Int., intestinal. Prog., progenitor. (**C**) UMAP visualization of the *Epcam*^+^ cells in wild-type (WT), *Lgr5^EGFP-IRES-CreERT2^*; *Apc*^Δ/Δ^; Rosa26^LSL*TdTomato*^ (LApc), and *Lgr5^EGFP-IRES-CreERT2^*; *Apc*^Δ/Δ^; *Tcf7*^Δ/Δ^; Rosa26^LSL*TdTomato*^ (LApcT) samples. The tumor-specific clusters are encircled. TA, transit-amplifying. EEC, enteroendocrine. (**D**) UMAP visualization showing reclustering of the tumor-specific cells in the LApc and LApcT merged samples and visualization of the clusters enriched (red color) in the LApcT samples. (**E**) UMAP visualization showing the reclustering of the tumor-specific cells in the LApc and *Lgr5^EGFP-IRES-CreERT2^*; *Apc*^Δ/Δ^; *Lef1*^Δ/Δ^; Rosa26^LSL*TdTomato*^ (LApcLef1) merged samples and visualization of the clusters enriched (blue color) in the LApcLef1 samples. (**F**) Normalized enrichment scores of canonical, noncanonical, and oncofetal gene sets analyzed using GSEA and fgsea R package applied to the cluster 1 cells in the LApc and LApcT samples. Presented are gene sets with padj. < 0.05 (table S3). (**G**) Enrichment scores for the noncanonical and oncofetal gene signatures [squamous (Squ.), endoderm (End.), epithelial-mesenchymal transition (EMT), injury repair (Inj.), neuroendocrine (Neu.), and oncofetal (Onc.)] in the cluster 1 cells (table S3). padj. values were quantified with the Benjamini-Hochberg false discovery rate (FDR) correction. (**H**) Normalized enrichment scores of canonical, noncanonical, and oncofetal gene sets analyzed with GSEA and fgsea R package applied to the cluster 1 cells in the LApc and LApcLef1 samples. Presented are gene sets with padj. < 0.05 (table S3). (**I** and **J**) UMAPs showing annotated cluster identities in the LApc, LApcT, and LApcLef1 samples (table S3). (**K** and **L**) UMAPs showing original annotation of the tumor-specific cells in the LApc, LApcT, and LApcLef1 samples.

To analyze plasticity in early-stage tumors of the large intestine, we reanalyzed published single-cell RNA sequencing (scRNA-seq) data from healthy individuals and from patients with FAP or colon carcinoma (GSE201348) (*8*, *27*). Upon clustering of the epithelial cells, we found a tumor-specific cluster, which was shared by both the FAP-polyp and carcinoma samples (Fig. 1A, fig. S1, A and B, and table S1). As expected, cells in this cluster showed high expression of *PROX1*, but unexpectedly, they also expressed multiple translineage plasticity markers, reflecting their HPCS (Fig. 1B).

To study plasticity in preclinical mouse models of early-stage intestinal adenomas, we induced *Apc* deletion in Lgr5^+^ cells by administering tamoxifen to *Lgr5^EGFP-IRES-CreERT2^*; *Apc^fl/fl^*; Rosa26^LSL*TdTomato*^ (LApc) mice (fig. S2A). Furthermore, to investigate the impact of Wnt/β-catenin signaling loss, we also induced *Tcf7* deletion by tamoxifen to *Lgr5^EGFP-IRES-CreERT2^*; *Apc^fl/fl^*; *Tcf7*^*fl/fl*^; Rosa26^LSL*TdTomato*^ (LApcT) mice (fig. S2A). Deletion of *Tcf7* was confirmed by immunofluorescence staining of the Tcf1 protein encoded by *Tcf7* gene in the tumor sections 19 days after tamoxifen administration (fig. S2B). To analyze tumor transcriptomics, we isolated intestinal tdTomato^+^ cells from these mice and subjected them to scRNA-seq (table S2). Furthermore, we reanalyzed our previously published scRNA-seq dataset (GSE179483) (*28*) containing tumor samples from LApc mice and mice deleted for both *Apc* and *Lef1* (*Lgr5^EGFP-IRES-CreERT2^*; *Apc*^Δ/Δ^; *Lef1*^Δ/Δ^; Rosa26^LSL*TdTomato*^) (LApcLef1) (fig. S3A). To identify tumor-specific cell clusters, we integrated intestinal epithelial samples from wild-type (WT) mice to the analysis (GSE169197) (*29*). After filtering and unsupervised clustering, Epithelial cell adhesion molecule-positive (*Epcam*^+^) cells were reclustered, and the clusters were annotated (Fig. 1C and figs. S2, C to E, and S3, B to D). *Tcf7* and *Lef1* deletions were associated with increased proportions of tumor-specific clusters, which was in line with the increased tumor burden observed in the LApcT and LApcLef1 mice compared to LApc mice (0.5, 5, and 36% for WT, LApc, and LApcT, respectively; 1, 14, and 56% for WT, LApc, and LApcLef1, respectively) (Fig. 1C, figs. S2, C and E, and S3, B and C, and table S3).

To investigate plasticity in cells of the tumor-specific clusters, we subsetted and reclustered them. We found that 5 out 12 and 7 of 13 clusters were present predominantly in the LApcT and LApcLef1 samples, respectively, indicating notable transcriptional changes in the *Tcf7*- and *Lef1*-deleted tumor cells (Fig. 1, D and E, and table S3). To understand the transcriptomic signatures of each cluster, we identified differentially expressed genes (DEGs) between the cell clusters separately for the LApc, LApcT, and LApcLef1 samples and performed gene set enrichment analysis (GSEA) with canonical intestinal epithelial and translineage plasticity gene signatures. In line with the human tumor data, all mouse tumor samples, including the LApc samples, showed enrichment of multiple translineage plasticity–associated gene signatures and repression of canonical intestinal epithelial signatures in cells of the cluster number 1 (Fig. 1, F to H, and fig. S4, A and B). Moreover, both LApcT and LApcLef1 samples presented multiple cell populations with high oncofetal and noncanonical plasticity signatures (Fig. 1, I and J). To further explore the cells that formed the highly plastic cluster 1, we set the identity back to the original annotation (Fig. 1C) and found that *Prox1*^+^ cells established this cluster in all tumor samples (Fig. 1, K and L, and figs. S2D and S3D). To further characterize changes in the transcriptional cell states following *Tcf7* or *Lef1* deletion, we performed TissueEnrich analysis on the DEGs in the tumor-specific cells between the *Tcf7*- or *Lef1*-deleted and control tumors. We found enrichment of genes associated with squamous epithelia, including skin and esophagus, and loss of genes associated with intestinal tissues, such as small intestine, stomach, duodenum, and colon in the deleted tumor samples (fig. S4, C and D). Together, we found a subpopulation of tumor cells with PCS in both intestinal human polyps and mouse adenomas and that *Tcf7* or *Lef1* deletion drives progressive plasticity in intestinal adenomas.

### *Tcf7* or *Lef1* deletion in intestinal adenomas boosts translineage plasticity

**Fig. 2.**
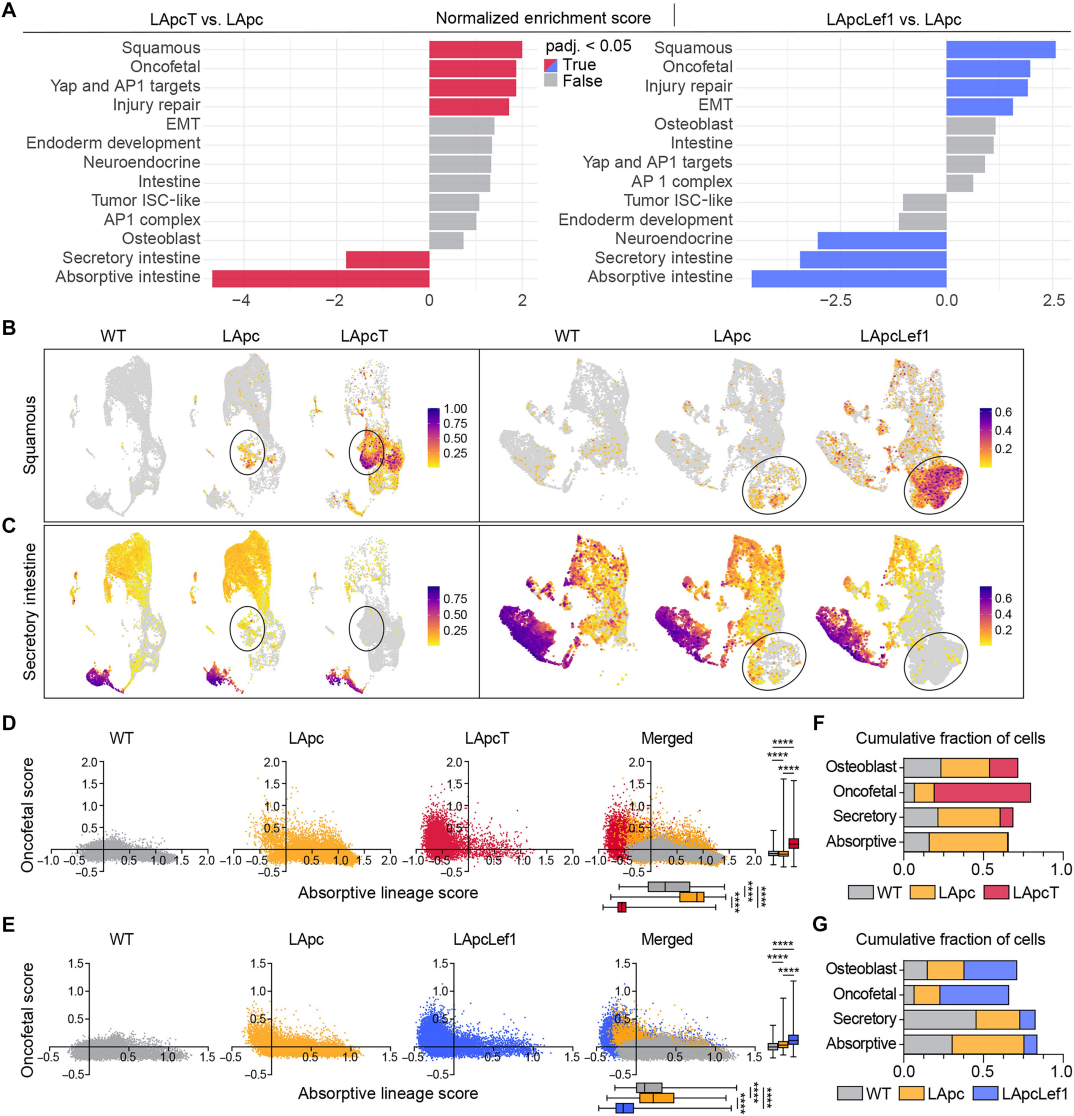
Deletion of either *Tcf7* or *Lef1* leads to loss of the canonical intestinal epithelial cell state. (**A**) Normalized enrichment scores of canonical, noncanonical, and oncofetal gene sets analyzed with GSEA and fgsea R packages in the LApcT or LApcLef1 versus LApc samples. Gene scores with padj. < 0.05 are presented in red or blue, and gene scores with padj. > 0.05 in gray. For statistical analysis, Benjamini-Hochberg FDR correction was used. (**B** and **C**) Module enrichment score (MES) plots showing the expression of (B) squamous hits (*Myl3*, *Lmo1*, *Egflam*, *Frmpd1*, *Hes5*, and *Atg9b*) and (C) secretory intestine gene signatures. The tumor-specific clusters are encircled. Color scale indicates the MES levels. Statistical significances were quantified with one-way analysis of variance (ANOVA) and Tukey post hoc test with the Benjamini-Hochberg method and are presented in table S3. (**D** and **E**) Scatter plots showing cell state distribution along the absorptive-oncofetal spectrum across the WT (gray), LApc (orange), LApcT (red), and LApcLef1 (blue) samples. Statistical tests used: one-way ANOVA and Tukey’s multiple-comparison test. ****P < 0.0001. (**F** and **G**) Distribution of module proportions in the WT (gray), LApc (orange), LApcT (red), and LApcLef1 (blue) samples. The module labels are based on the >0.75 quantile score for each cell and gene module (Materials and Methods).

As translineage plasticity can influence all tumor cells and not just tumor-specific cells, we returned to analyze the dataset comprising all tumor cells. In addition to analyzing canonical intestinal epithelial and translineage plasticity gene signatures (table S3), we also included a list of Yes-associated protein 1 (Yap1) target genes and genes encoding Activator protein 1 (AP-1) subunits and their target genes, which have been shown to control oncofetal reprogramming (*9*). GSEA of the DEGs revealed decreased expression of canonical intestinal epithelial gene sets and enriched expression of oncofetal and noncanonical plasticity gene sets in both *Tcf7*- or *Lef1*-deleted tumor cells (Fig. 2, A to C). To explore this in more detail, we calculated the expression of these gene modules for each cell in each treatment group and plotted the values. Although *Apc* deletion increased expression of the oncofetal gene signature and decreased the absorptive gene signature compared to the WT samples, additional deletion of *Tcf7* or *Lef1* substantial boosted these effects (Fig. 2, D to G). These results indicate that the deletion of either *Tcf7* or *Lef1* boosts translineage plasticity. Moreover, we analyzed expression of genes known to be either up-regulated or down-regulated 24 hours after diphtheria toxin induced loss of Lgr5^+^ tumor cells (*20*). A gene signature for genes up-regulated upon loss of the Lgr5^+^ cells was enriched in both LApcT and LApcLef1 samples compared to their control tumors (table S3). In addition, a gene signature for genes down-regulated upon loss of Lgr5^+^ cells was enriched in the LApcT tumor samples (table S3). Thus, these results suggest that genes activated upon loss of the Lgr5^+^ tumor cells are also activated upon *Tcf7* or *Lef1* deletion–induced progressive plasticity.

To explore whether samples from patients with CRC and low canonical WNT signaling score show a high transplasticity score, we explored these correlations in microarray data derived from patients with CRC (GSE39582) (*30*). As expected, we found the highest canonical intestinal epithelial score (HiCol score: 10) in patient samples with the highest WNT score (fig. S5A). In contrast, the highest squamous score (HiSquam score: 10) was found in cells with the lowest WNT score (fig. S5B). In line with this, samples with the lowest *TCF7* expression also showed the lowest canonical intestinal epithelial score and the HiSquam score (fig. S5, C and D). However, no correlation was found between *LEF1* expression and canonical intestinal epithelial or squamous scores (fig. S5, E and F). Together, we found a negative correlation between WNT/*TCF7* and the transplasticity squamous signature also in samples from patients with CRC.

### Genes encoding AP-1 subunits are activated following *Tcf7* or *Lef1* deletion

**Fig. 3.**
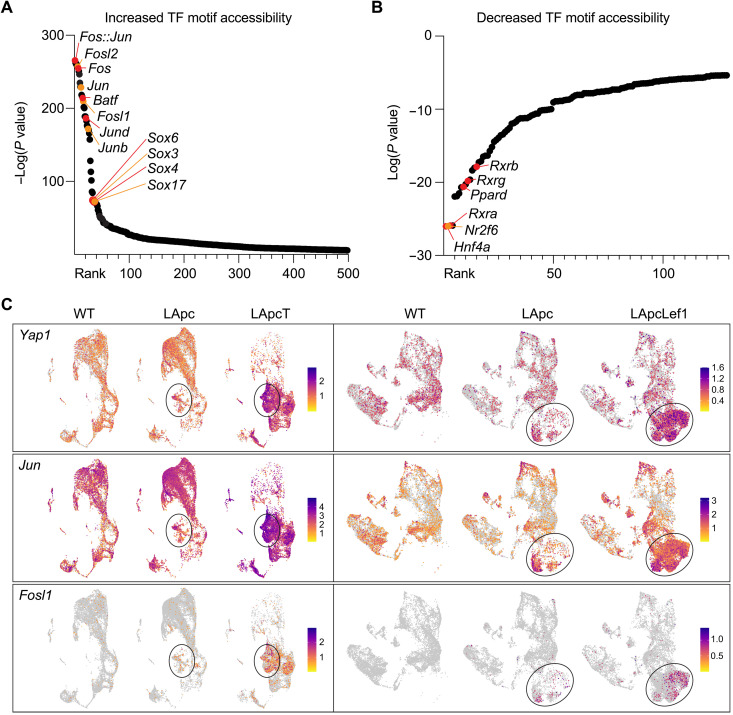
Modulation of chromatin accessibility and expression of subunits of the AP-1 complex in the *Tcf7-* or *Lef1*-deleted *Apc* mutant tumor cells. (**A** and **B**) Tdtomato^+^ fluorescence-activated cell sorting (FACS)–sorted tumor cells from four LApc and four LApcLef1 mice were subjected to ATAC-seq. Enrichment of transcription motifs (TF) for (A) more and (B) less accessible regions was analyzed with simple enrichment analysis (SEA; Materials and Methods). Elbow plots showing TF activity in LApcLef1 versus LApc tumor samples. Table S3 shows the *P* values for the presented genes from top rank to lowest. (**C**) Feature plots showing the expression of *Yap1*, *Jun*, and *Fosl1* in the indicated samples. The color scale indicates gene expression levels. Statistical significances were quantified with one-way ANOVA and Tukey post hoc test with the Benjamini-Hochberg method and are presented in table S3.

To explore how oncofetal programming is affected by deletion of Wnt effectors, we analyzed chromatin accessibility in LApcLef1 and LApc tumors using assay for transposase-accessible chromatin sequencing (ATAC-seq). We found that *Lef1* deletion increased chromatin accessibility at the transcription factor (TF) motifs of AP-1 subunits’ *Fos*, *Fosl2*, *Jun*, *Batf*, *Fosl1*, *Jund*, and *Junb*, which are drivers of oncofetal reprogramming (*9*), and multiple Sox family members, including *Sox17* (Fig. 3A). We also found decreased chromatin accessibility at the TF motifs of *Hnf4a*, *Nr2f6*, *Rxra*, *Ppard*, and *Rxrg*, which act as protectors of canonical intestinal epithelial features (Fig. 3B) (*9*). Furthermore, we explored published TF motif enrichment analysis of ATAC-seq data from patients with CRC submitted to The Cancer Genome Atlas (*10*) and found loss of accessibility at sites that contained motifs for *TCF7* and *LEF1* in patients with a high squamous gene signature, thus supporting our findings.

To investigate whether changes in chromatin accessibility of TF motifs correlate with altered RNA expression, we analyzed scRNA-seq data from the WT, LApc, LApcT, and LApcLef1 samples. In line with the ATAC-seq results, several oncofetal markers, including, e.g., *Yap1*, *Anxa1*, *Fos*, *Fosl1*, and Sox family members (e.g., *Sox4*, *Sox13*, and *Sox17*), were more highly expressed in LApcT and LApcLef1 cells than in LApc tumor cells (Fig. 3C and fig. S6). However, despite the decreased chromatin accessibility of their TF motifs, the *Hnf4a*, *Nr2f6*, *Rxra*, *Ppard*, and *Rxrg* transcripts were not decreased in LApcT or LApcLef1 versus LApc samples (fig. S7A). Overall, these results further indicated that *Tcf7* and *Lef1* suppress progressive plasticity in intestinal adenomas.

As subunits of the AP-1 complex also function as effectors of noncanonical Wnt/planar cell polarity (PCP) signaling pathway (*31*), we investigated whether deletion of *Tcf7* or *Lef1* drives expression of additional noncanonical Wnt signaling components belonging to Wnt/PCP or Wnt/Ca^2+^ pathway. In the tumor scRNA-seq data, module enrichment score (Materials and Methods) for noncanonical Wnt was increased especially in the tumor-specific cell clusters upon *Tcf7* or *Lef1* deletion (fig. S7B). These results suggest increased activation of the noncanonical Wnt signaling in association with progressive plasticity.

### *Kras* mutation suppresses Lef1 and Prox1 expression in intestinal adenomas

It has been suggested that in CRC, suppression of the canonical Wnt-high tumor state enables oncofetal reprogramming, leading to a noncanonical cell state that drives CRC progression (*9*). Although nongenetic and genetic drivers for such reprogramming have been reported (*8*–*10*), the mechanisms initiating plasticity reprogramming are incompletely understood. As loss-of-function mutations in the WNT effectors *TCF7* and *LEF1* are rare in patients with CRC (*8*), loss of canonical WNT pathway activity must be driven by other factors. *Kras* mutations have been shown to switch canonical Wnt activation from a hyperactive to a hypoactive state in preclinical intestinal tumor models (*32*). To assess whether mutant *Kras* affects the expression of T cell factor 1 (Tcf1) and Lef1, we stained them in tumor sections from *Apc^Min/+^* and *Apc^Min/+^; Kras^LSL-G12D/+^*; *Villin*-Cre^ERT2^ mice (fig. S8A). We also stained the sections for Prox1, which is a Wnt target gene highly expressed by *Apc* mutant CRC tumors (*21*, *33*) and which is expressed by *Apc^Min/+^* and CRC tumor stem cells (*22*, *23*). In line with the hypoactive canonical Wnt signaling state following *Kras* mutation, we found decreased expression of Lef1 and Prox1, but not Tcf1, in the *Kras* mutant tumors (fig. S8, B to G). To further evaluate this in samples from patients with CRC, we analyzed microarray data from samples of patients with Consensus molecular subtyped (CMS-subtyped) CRC (GSE39582) (*30*). We found that in the CMS3-subtype tumors that are characterized by *KRAS* mutation, *LEF1* expression was lower than in the other CMS subtypes (*28*, *34*). In addition, *TCF7* expression was lower in the CMS3 samples when compared to Wnt-high CMS2 samples (fig. S8H). In summary, our results from mouse tumors and data from patients with CRC indicate that *KRAS* mutations indeed drive a hypoactive canonical Wnt state in CRC.

### Prox1 expression is decreased upon *Tcf7* or *Lef1* deletion

**Fig. 4.**
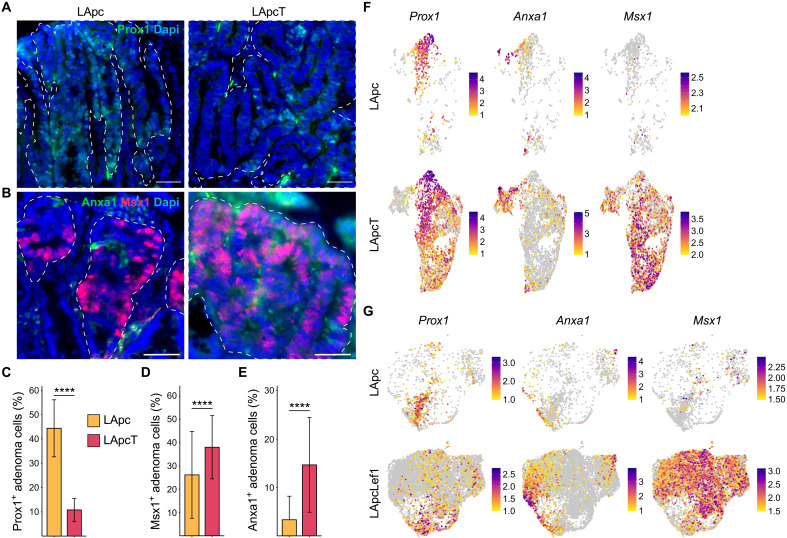
Validation of oncofetal and osteoblast reprogramming in *Tcf7*- or *Lef1*-deleted tumor cells. (**A** and **B**) Immunofluorescence staining of (A) Prox1^+^ (green) and (B) Anxa1^+^ (green) together with Msx1^+^ (red) tumor cells in the LApc and LApcT samples 19 days after tamoxifen administration. The dashed lines indicate the nuclear β-catenin^+^ tumor areas. Scale bars, 50 μm. (**C** to **E**) Quantification of the (C) Prox1^+^ (number of mice: LApc: 4, LApcT: 4; number of quantified tumors: LApc: 25, LApcT: 26; ****P = 3.8 × 10^−18^, using Student’s *t* test), (D) Msx1^+^, and (E) Anxa1^+^ (number of mice: LApc: 7, LApcT: 7; number of quantified tumors: LApc: 57, LApcT: 59; ****P = 0.00015, ****P = 2.4 × 10^−12^, using Student’s *t* test) tumor cells in the LApc (orange) and LApcT (red) samples. Average ± SD values are shown in the bar plots. (**F**) Feature plots showing the expression of *Prox1*, *Anxa1*, and *Msx1* in LApc (top panel) and LApcT (bottom panel) samples. The color scale indicates gene expression levels. (**G**) Feature plots showing the expression of *Prox1*, *Anxa1*, and *Msx1* in LApc (top panel) and LApcLef1 (bottom panel) samples. (F and G) Statistical significances were quantified with one-way ANOVA and Tukey post hoc test with the Benjamini-Hochberg method and are presented in table S3.

Although previous data from preclinical mouse intestinal tumor model indicated a prometastatic function for Prox1 (*35*), another study showed an increase of translineage plasticity markers following *PROX1* silencing in organoids derived from patients with CRC (*8*). To study the effects of *Tcf7* or *Lef1* deletion on Prox1 expression, we stained mouse tumor sections for Prox1 and for annexin A1 (Anxa1), a marker of oncofetal reprogramming and a gene negatively controlled by Prox1 (*9*, *22*), and for Msh homeobox 1 (Msx1), a noncanonical osteoblast marker (*8*). Similarly, as in the *Lef1*-deleted samples (*28*), we found strongly decreased Prox1 and increased Msx1 and Anxa1 expression in the *Tcf7*-deleted tumors (Fig. 4, A to E, and table S4). While Anxa1^+^ cells were mainly negative for Prox1 in the stained tumor sections and in the scRNA-seq analysis of the cells in the tumor-specific clusters, the *Msx1*^+^ tumor cells were strongly *Prox1*^+^ and mainly *Anxa1*^−^ in the scRNA-seq data. *Msx1* expression was found mainly in the *Anxa1*^−^ tumor–specific cells (Fig. 4, F and G). Together, these results indicate that deletion of either *Tcf7* and *Lef1* suppresses Prox1 expression and suggest that oncofetal and osteoblast-like cells marked by Anxa1^+^ and Msx1^+^, respectively, represent distinct tumor cell populations.

### Inhibition of Lgr5^+^ tumor stem cell lineage commitment upon progressive plasticity

**Fig. 5.**
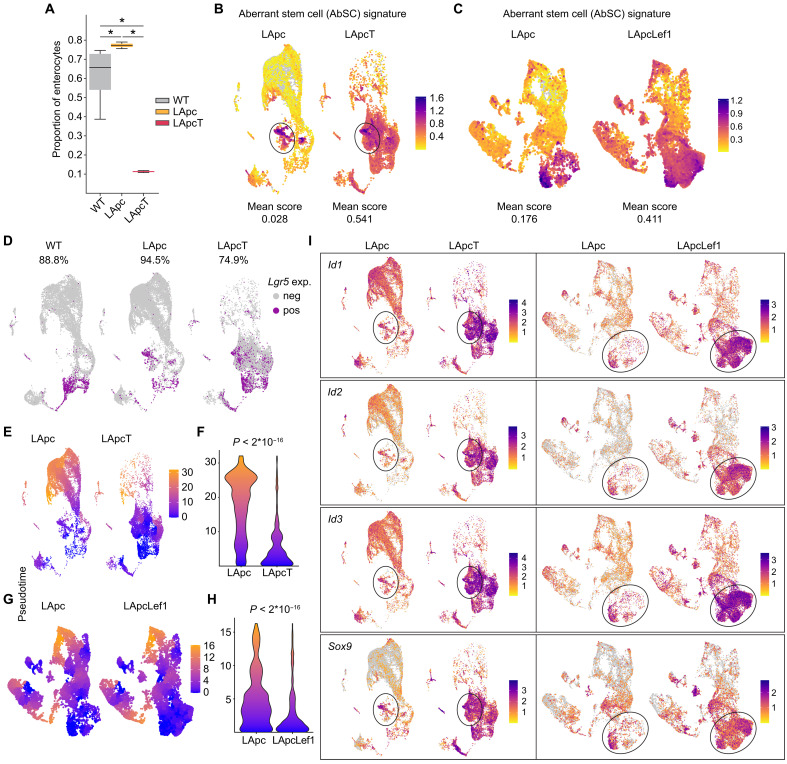
Differentiation of Lgr5^+^ tumor cells is inhibited in *Tcf7*- or *Lef1*-deleted tumors. (**A**) Proportions of cells in the enterocyte clusters from the indicated samples. Credible and significant are denoted by * (FDR < 0.05). (**B** and **C**) MES plots showing the expression of the aberrant stem cell (AbSC) signature (*26*). The color scale indicates the MES levels. Statistical significances were quantified with one-way ANOVA and Tukey post hoc test with the Benjamini-Hochberg method and are presented in table S3. (**D**) Feature plots showing Lgr5^+^; tdTomato^+^ cells (purple) and Lgr5^−^; tdTomato^+^ cells (gray) in the WT, LApc, and LApcT samples. The mean proportions of Lgr5^−^; tdTomato^+^ cells per treatment group are marked under the sample names. neg, negative. pos, positive. (**E** to **H**) Trajectory analysis was performed with Monocle3. (E) Feature and (F) violin plots of the pseudotimes in the LApc and LApcT samples; (G) feature and (H) violin plots of the pseudotimes in the LApc and LApcLef1 samples. Statistical significances were quantified with one-way ANOVA and Tukey post hoc test with the Benjamini-Hochberg method. The color scale indicates pseudotimes. (**I**) Feature plots showing the expression of *Id1-3* and *Sox9*. The color scale indicates gene expression levels. Statistical significances were quantified with one-way ANOVA and Tukey post hoc test with the Benjamini-Hochberg method and are presented in table S3.

We have previously reported that Lgr5^+^ cells in *Apc^Min/+^* tumors differentiate predominantly into enterocyte-like tumor cells (*23*). Cell type proportion analysis of the tumor samples showed a decreased enterocyte-like cell population both in the *Tcf7*- or *Lef1*-deleted tumors, suggesting intralineage plasticity and blocked differentiation (78 and 11% in LApc versus LApcT analysis, respectively; 35 and 12% in LApc versus LApcLef1 analysis, respectively) (Fig. 5A). Intestinal differentiation correlates negatively with an aberrant stem cell score in CRC (*26*, *36*). Thus, we quantified this score using module enrichment (Materials and Methods) and found that it was increased in the *Tcf7*- or *Lef1*-deleted samples compared to the control samples, suggesting reduced tumor cell differentiation (Fig. 5, B and C). As our LApcT analysis includes a tdTomato-lineage tracer and as the deletions were induced in Lgr5^+^ cells, we evaluated the proportion of Lgr5^−^; tdTomato^+^ daughter cells within the tumor cell population. We found a 20% decrease in Lgr5^−^; tdTomato^+^ progeny cells in the LApcT samples compared to the LApc samples, providing additional evidence for the inhibition of tumor cell differentiation (Fig. 5D). To further analyze changes in tumor cell trajectories, we analyzed pseudotime using the Monocle3 package (RRID:SCR_018685) (*37*–*40*) and found a higher proportion of cells with a low pseudotime value in the LApcT or LApcLef1 samples compared to the LApc tumors (Fig. 5, E to H). To find an explanation for the inhibited differentiation, we analyzed DEGs between the LApc and LApcT or LApcLef1 samples. Among the up-regulated DEGs in *Tcf7*- or *Lef1*-deleted samples, we found transcripts encoding genes that function as inhibitors of tumor cell differentiation, including, e.g., inhibitors of differentiation 1, 2, and 3 (*Id1/2/3*) and Sox9 (*25*, *26*). The expression of these genes was increased in both LApcT and LApcLef1 samples, especially in the tumor-specific clusters, and in stem and progenitor cells (Fig. 5I). Together, these results provide evidence of impaired tumor cell differentiation of the Lgr5^+^ tumor stem cells upon progressive intralineage plasticity.

To analyze the enterocyte-like tumor cell population in tumor samples isolated from the patients, we quantified enterocyte-like cell proportions from healthy tissue, FAP polyp, and carcinoma samples (Fig. 1A and fig. S1B). Moreover, we analyzed DEGs between the polyps and carcinoma samples. As expected, many of the translineage plasticity programs were enriched in the carcinoma samples compared to the polyps, reflecting disease progression. Moreover, in line with our mouse adenoma data, the total enterocyte-like cell population was decreased in the polyp and carcinoma samples compared to the healthy tissue samples, but not between the polyp and carcinoma samples (fig. S1B). However, *EMP1* positivity increased upon disease progression, as 63 and 92% of the enterocyte-like cells expressed *EMP1* in the polyp and carcinoma samples, respectively. This is of interest, as *EMP1* is associated with plasticity and a poor prognosis in patients with CRC (*19*, *41*).

### Plasticity-associated increase in tumor cell proliferation and tumor growth is Myc dependent

**Fig. 6.**
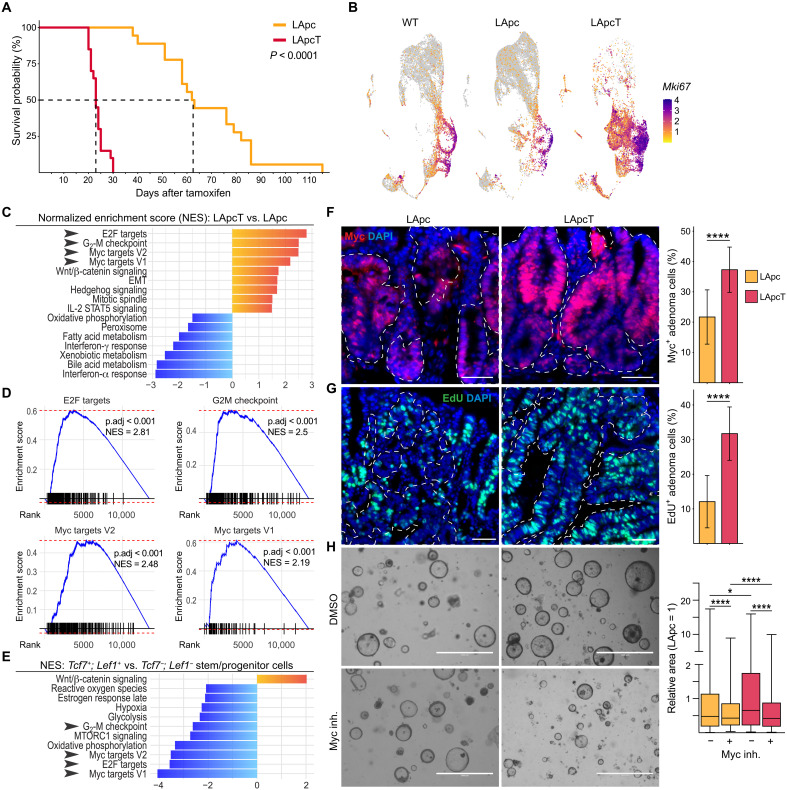
Inhibition of Myc repeals *Tcf7* deletion–induced organoid growth. (**A**) Kaplan-Meier survival curves of the LApc (*n* = 18) and LApcT (*n* = 20) mice. Probabilities were quantified with survival (*68*) and survminer (*69*) (log-rank test). (**B**) Feature plots of *Mki67*. Color scale indicates gene expression (one-way ANOVA and Tukey post hoc test with the Benjamini-Hochberg method; table S3). (**C**) Normalized enrichment score (NES) of Hallmark pathways quantified with GSEA and MSigDB and fgsea, based on the DEGs between the LApcT and LApc cells. Pathways with padj. < 0.05 are shown (Benjamini-Hochberg FDR correction; table S3). IL-2, interleukin 2; STAT5, signal transducer and activator of transcription 5. (**D**) Enrichment scores for E2F targets, G_2_-M checkpoint, and Myc targets V1 and V2. (**E**) NES of Hallmark pathways (presented are pathways with scores <−2 or ≥2, and padj. < 0.05) quantified with GSEA and MSigDB and fgsea R packages, based on the DEGs between the *Tcf7*^+^; *Lef1*^+^ stem cells versus *Tcf7*^−^; *Lef1*^−^ in the LApc mice. MTORC1, Mechanistic target of rapamycin complex 1. (**F** and **G**) Immunofluorescence stainings and quantifications of (F) Myc^+^ (red) (number of mice: LApc: 13, LApcT: 12; number of quantified tumors: LApc: 55, LApcT: 71; ****P = 3.1 × 10^−19^; Student’s *t* test) and (G) EdU^+^ (green) (number of mice: LApc: 12, LApcT: 12; number of quantified tumors: LApc: 81, LApcT: 113; *P = 3.8 *10^−18^; Student’s *t* test) in tumor cells in the LApc and LApcT samples. Dashed lines indicate the nuclear β-catenin^+^ tumor areas. Scale bars, 50 μm. Average ± SD values are presented. (**H**) Representative bright-field images of the treatment groups and quantifications of relative organoid area [LApc with dimethyl sulfoxide (DMSO) treatment = 1]. Organoids were isolated from 3 + 3 mice, and this was repeated three times. Number of organoids quantified: LApc: 699; LApc Myc inhibitor: 1118; LApcT: 913; LApcT Myc inhibitor 1412. *P = 0.0207 and ****P < 0.0001 (two-way ANOVA and Tukey’s multiple-comparison test). Scale bars, 1000 μm. inh, inhibitor. c(EN4) = 50 μM.

To investigate whether *Tcf7* deletion–induced progressive plasticity is associated with enhanced tumorigenesis similarly as following *Lef1* deletion (*28*), we performed survival analysis on the LApcT and LApc mice. *Tcf7* deletion led to markedly decreased survival of the LApcT mice compared to the LApc mice, resulting in average life spans of 24 and 68 days, respectively (Fig. 6A), thus confirming that also *Tcf7* inhibits tumorigenesis in *Apc* mutant mice. This finding was supported by increased *Mki67* expression and the proportion of proliferating cells in the *Tcf7*-deleted samples (16, 6, and 32% in WT, LApc, LApcT samples, respectively) (Fig. 6B). Correspondingly, in the WT, LApc, and LApcLef1 samples, the proportions of proliferating cells were 19, 16, and 49% (fig. S3C). Furthermore, GSEA of the DEGs revealed enhanced Myc, E2F, and G_2_-M pathways in the *Tcf7*-deleted tumors (Fig. 6, C and D). In line with this, by analyzing *Tcf7*^+^; *Lef1*^+^ double-positive and *Tcf7*^−^; *Lef1*^−^ double-negative stem cells in the control LApc tumors, we found suppressed Myc activity in the double-positive cells (Fig. 6E). Moreover, we observed increased Myc immunofluorescence staining and 5-ethynyl-2′-deoxyuridine (EdU) incorporation in the LApcT tumor sections compared to the LApc samples, similarly as reported previously for the LApcLef1 samples (Fig. 6, F and G) (*28*). To confirm that Myc was involved in the increased tumorigenesis, we isolated organoids from LApc and LApcT mice 29 and 19 days after in vivo gene deletions, respectively. As expected, the average size of the *Tcf7*-deleted organoids was larger than that of control organoids (Fig. 6H). However, this difference was abolished by treatment with the EN4 Myc inhibitor (Fig. 6H) (*42*). These results indicate a vital role of Myc in promoting tumor growth upon progressive plasticity.

### High *TCF7* expression attenuates the adverse prognostic impact of translineage plasticity 

**Fig. 7.**
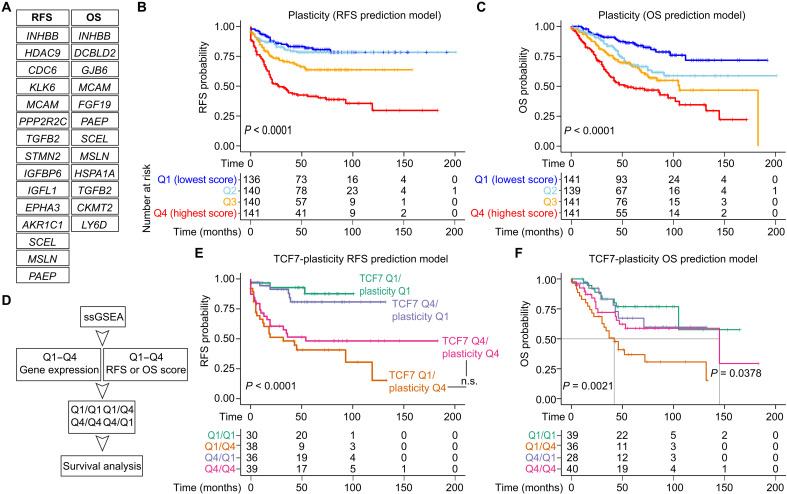
*TCF7* expression correlates with better OS in patients with CRC with high plasticity score. (**A** to **F**) GSE39582 containing microarray data from samples of patients with CRC was reanalyzed. (A) Final plasticity gene signatures for RFS and OS. (B and C) RFS (B) and OS (C) probability was quantified for Q1 (<0.25 percentile, lowest plasticity score, blue line), Q2 (light blue line), Q3 (orange line), and Q4 (>0.75 percentile, highest plasticity score, red line). (D to F) Schematic (D) of the analysis for (E) and (F). First, RFS and OS plasticity scores for each patient sample were quantified with single-sample (ss) GSEA and gene set variation analysis (GSVA) R package, and samples were divided into four groups based on the score (Q1 to Q4). Then, patient samples were also divided into four groups (Q1 to Q4) based on their *TCF7* expression. For the survival analysis, we selected samples with highest (Q4) and lowest (Q1) *TCF7* score and highest (Q4) and lowest (Q1) plasticity score. We compared RFS (E) and OS (F) probabilities between Q1 (lowest *TCF7*)–Q1 (lowest plasticity score) (green line), Q1-Q4 (orange line), Q4-Q4 (pink line), and Q4-Q1 (purple line). In (F), gray lines present 50% survival probability. Probabilities were quantified in (B) and (C) and (E) and (F) with survival (*68*) and survminer (*69*) R packages. Statistical significances were quantified with Cox proportional hazards regression model. n.s., not significant.

Although individual translineage plasticity programs are associated with tumor recurrence and survival in patients with CRC (*8*), a possible correlation between oncofetal signatures and survival is unknown. In addition, a general translineage plasticity gene signature with a limited number of genes derived from oncofetal and noncanonical plasticity markers is lacking. To identify prognostic genes associated with recurrence-free survival (RFS) and overall survival (OS), we reanalyzed a published cohort of patients with CRC (GSE39582) (*30*) and performed a multistep analysis combining gene expression data with clinical outcomes (Materials and Methods). This enabled us to generate translineage plasticity and oncofetal signatures for the RFS and OS analyses (Fig. 7A). We found that translineage plasticity and oncofetal signatures were negatively correlated with both RFS and OS, as the worst survival was found in patients with the highest translineage plasticity or oncofetal score (Fig. 7, B and C, and fig. S9, A and B).

Moreover, we analyzed whether *TCF7* or *LEF1* expression together with the translineage plasticity score correlates with patient survival. To do this, we stratified the samples based on their quartile gene expression and translineage plasticity score, and based on this, we assigned to each sample a combined label representing both its gene expression and translineage plasticity score quartiles (e.g., Q1_Q1; Materials and Methods and Fig. 7D). Although *LEF1* expression did not correlate with RFS or OS (fig. S9, C and D), Kaplan-Meier survival analysis for *TCF7* showed that patients in the Q1_Q4 group, characterized by the lowest *TCF7* expression and highest translineage plasticity score, had the poorest OS and a trend of worse RFS (Fig. 7, E and F).

## DISCUSSION

**Fig. 8.**
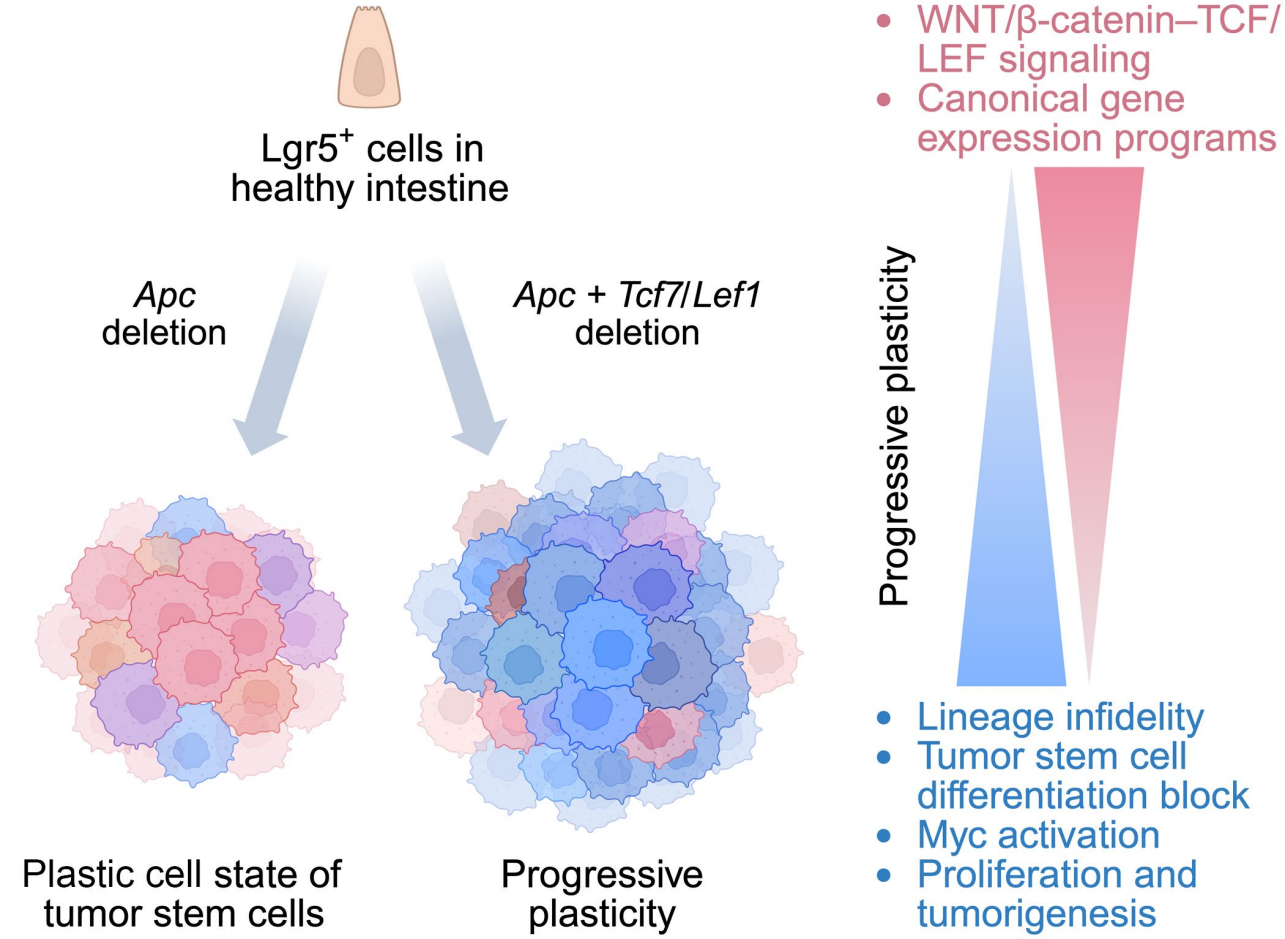
A schematic summary of the main results. *APC* mutations in intestinal tumorigenesis are known to cause hyperactivation of WNT/β-catenin signaling that results in epigenetic rewiring. We show that tumor stem cells in intestinal polyps and adenomas exhibit an PCS. Furthermore, deletion of *Tcf7* or *Lef1* gene in mouse intestinal adenomas promotes progressive plasticity, as evidenced by tumor stem cell differentiation block, cell lineage infidelity, increased expression of translineage plasticity markers, plus activation of Myc, which enhances tumor cell proliferation and tumorigenesis. Created in BioRender. M.L. (2026); https://BioRender.com/vgedehu.

Here, we show PCS in *PROX1*^+^ tumor stem cells in human colorectal polyps and mouse intestinal adenomas, indicating that translineage plasticity is associated with early CRC development. Furthermore, homozygous deletion of the Wnt signaling mediator *Tcf7* or *Lef1* together with *Apc* deletion in Lgr5^+^ cells resulted in progressive plasticity of the adenoma cells. Deletion of *Tcf7* or *Lef1* in Lgr5^+^ cells resulted in loss of their lineage commitment to enterocyte-like tumor cells reflecting intralineage plasticity. We also demonstrated that the enhanced tumorigenesis following these deletions is mediated by increased Myc signaling. Moreover, we show that patients with CRC with the lowest *TCF7* expression and the highest translineage plasticity score have the worst OS. In summary, we show that plasticity is not only a feature of advanced CRCs but is present already in tumor stem cells of colorectal polyps and intestinal mouse adenomas and that progressive plasticity driven by loss of canonical Wnt results in failure of Lgr5^+^ tumor stem cell differentiation and enhanced Myc-dependent tumorigenesis (Fig. 8).

Although pathological activation of the Wnt/β-catenin signaling is the most common initiating event in patients with CRC (*11*, *12*, *14*), we show here that Wnt/β-catenin signaling via *Tcf7* and *Lef1* restricts tumor cell proliferation. Deletion of *Tcf7* or *Lef1* in *Apc* mutant tumor cells resulted in robustly increased tumor cell proliferation, which led to a shorter life span of the mice. Both gene deletions led to decreased canonical Wnt target gene expression, which confirmed the conversion from a canonical Wnt-hyperactive state to a canonical Wnt-hypoactive state. However, we also found increased expression of noncanonical Wnt/Ca^2+^ and Wnt/PCP pathway components upon *Tcf7* or *Lef1* deletion in scRNA-seq analysis, suggesting enhanced noncanonical Wnt signaling upon progressive plasticity. Additional studies are needed to reveal the mechanisms involved in this activation and the functional outcome of the increased noncanonical Wnt signaling. The possible role of the noncanonical Wnt in progressive plasticity is intriguing, as it has been linked to invasion, survival, and metastasis (*31*, *43*, *44*).

So far, it has been unclear whether the plasticity process also affects tumor stem cells. A covariation between high *PROX1* expression and translineage plasticity markers has been reported by RNA sequencing analysis of two distinct cohorts of patients with CRC (*8*, *45*). However, silencing of *PROX1* resulted in increased expression of translineage plasticity markers in organoids derived from patients with CRC that had a strong canonical intestinal epithelial signature (*8*). Here, we show that *PROX1*^+^ *APC* mutant intestinal human polyp and mouse adenoma stem cells express multiple translineage plasticity markers indicating their PCS. In addition, we show decreased, but not completely abolished, Prox1 expression in *Tcf7*- or *Lef1*-deleted tumor cells. Moreover, the oncofetal marker *Anxa1* was expressed mainly in the *Prox1*^−^ cells. This is in line with our previous study describing increased Anxa1 expression following *Prox1* deletion or silencing in *Apc* mutant tumors and CRC cell lines and mutually exclusive staining of ANXA1 and PROX1 in CRC tissue sections (*22*). In contrast, tumor cells expressing the osteoblast marker *Msx1* showed high *Prox1* expression even in the *Tcf7*- or *Lef1*-deleted tumor samples. Collectively, we show that the *Anxa1*^+^ oncofetal and *Msx1*^+^ (and *Prox1*^+^) osteoblast–like cells are two distinct tumor cell populations, suggesting that Prox1 expression differs between distinct translineage plasticity programs. Thus, defining the context-dependent functions of Prox1 across Wnt-driven and noncanonical plasticity programs represents an important remaining challenge.

Previous studies have shown that the Lgr5^+^ tumor cells function as tumor stem cells (*15*–*17*, *23*, *46*). Although Lgr5^+^ stem cells give rise to multiple tumor cell lineages in *Apc^Min/+^* tumors, their progeny consists predominantly of enterocyte-like tumor cells (*23*). Consistent with this, deletion of *Tcf7* or *Lef1* in Lgr5^+^ cells resulted in a decreased proportion of *Lgr5*^−^; *tdTomato*^+^ progeny cells and enterocyte-like tumor cells, which was associated with increased expression of differentiation inhibitors, including, e.g., *Id1*-*Id3* and *Sox9* in the *Tcf7*- or *Lef1*-deleted tumors. Notably, no significant changes were found in the secretory cell populations upon *Tcf7* or *Lef1* deletion. Our results on the enterocyte-like cells agree with inhibition of differentiation as an initiating event preceding CRC development (*26*). Moreover, these results and our finding of increased oncofetal gene expression upon *Tcf7* or *Lef1* deletion are in line with published data showing that Sox9 mediates impaired intestinal epithelial differentiation and drives reactivation of fetal intestinal genes upon tumorigenesis (*26*, *36*). The transition between stemlike activity and differentiation in CRC has been shown to be controlled by epigenetic regulators [SMARCB1 of the BRG1/BRM-associated factor (BAF) complex], supporting our findings of modifications in the chromatin accessibility of both oncofetal drivers and intestinal epithelial protectors (*36*). A study describing the malignant transformation of polyps to carcinoma showed decreased enterocyte populations in patient samples (*27*). Our reanalysis including these samples and additional tumor samples confirmed this result. Overall, our results indicate that the canonical Wnt-dependent tumor state is required for Lgr5^+^ tumor stem cell differentiation into enterocyte-like cells and that both human and mouse tumor samples show decreased enterocyte-like tumor cell populations upon progressive intralineage plasticity.

The deletion of *Lef1* was associated with reduced chromatin accessibility at the TF motifs of multiple protectors of the intestinal epithelial lineage, for example, *Hnf4a*, *Rxra*, and *Ppard*. This result is in line with a published study showing decreased chromatin accessibility of these genes upon progressive plasticity (*9*). However, in the present study, these genes did not show decreased expression according to our scRNA-seq data. On the other hand, we found increased chromatin accessibility of the oncofetal TF motifs and expression of oncofetal genes, including, e.g., *Yap1* and genes encoding multiple AP-1 subunits in the *Tcf7*- or *Lef1*-deleted tumors, which is in line with published data (*9*). We also found increased chromatin accessibility of the TF motifs and the expression of translineage plasticity markers and multiple Sox family members, such as Sox9 and Sox17, following *Tcf7* or *Lef1* deletion. These data indicate that loss of canonical Wnt via deletion of either *Tcf7* or *Lef1* in *Apc* mutant tumor cells mimics the effects on chromatin accessibility that have been reported in more advanced preclinical mouse tumor models (*9*).

Although additional regulators of plasticity have been intensively investigated, their downstream effectors and druggable targets remain largely unknown. Here, we found increased Myc expression in the *Tcf7*-deleted tumors, which is in line with our previous results concerning *Lef1* deletion (*28*). We found that the increased organoid growth induced by *Tcf7* deletion was repressed by inhibition of Myc, which indicates that Myc has a vital role in tumor cell proliferation associated with progressive plasticity. This is consistent with a previous study showing increased Myc signaling after Lgr5^+^ cell depletion in *Apc^Min/+;^Lgr5^DTR/eGFP^;Villin^Cre^;Kras^LSL-G12D^* organoids (*20*). Moreover, expression of *Sox17*, a direct Myc target (*47*), was also increased in the *Tcf7*- or Lef1-deleted tumor samples (*28*). As Sox17 boosts tumor growth by mediating tumor immune evasion in early-phase CRC (*48*), further studies exploring the inhibition of tumor progression by targeting Myc signaling in the context of tumor cell plasticity and immune evasion will be of interest. Moreover, as Myc inhibition in healthy fetal intestinal organoids increased the proportion of enterocyte cells, additional studies are needed to explore whether Myc signaling could directly affect the loss of the enterocyte-like population upon progressive plasticity (*49*).

*TCF7* and *LEF1* mutations are rare in patients with CRC (*8*). However, as the *TCF7* gene is located close to the *APC* gene in humans (5q31.1 and 5q22.2, respectively), it may occasionally be included in the large deletions of chromosomal DNA that occur upon tumor initiation when the WT *APC* allele is lost. However, plasticity is thought to be driven mainly by epigenetic processes rather than mutations (*8*). TF motif enrichment analysis of ATAC-sequenced samples from patients with CRC revealed a loss of accessibility at sites that contained TF motifs for both *TCF7* and *LEF1* in patients with high squamous-like gene signature (*10*). This suggests that during noncanonical reprogramming in CRC, changes driving progressive plasticity occur in *TCF7* and *LEF1* accessible chromatin, supporting our data on the association between *Tcf7* or *Lef1* deletion and tumor cell reprogramming in *Apc* mutant adenomas. We found decreased Lef1 and Prox1 expression in *Kras* mutant *Apc^Min/+^* tumors, which is in line with a recent study (*32*) showing CRC conversion from a WNT-high to a WNT-low tumor status upon *Kras* mutation. According to this scenario, *KRAS* mutations, which are frequently found in patients with CRC, induce *LEF1* down-regulation, which, in turn, increases CRC progression by increasing tumor cell plasticity. These results are in line with a study describing increased *Tcf7* and *Lef1* mRNA expression upon chemical Kras^G12D^ inhibition, which also induced a conversion from *Emp1*^+^ plastic tumor cell population to canonical Wnt-driven *Lgr5*^+^ cell state (*19*). Recent studies have also demonstrated that CRC cells dynamically shift between a MAPK-driven state and a WNT-driven canonical state, and that MAPK inhibition promotes adoption of the latter phenotype (*50*, *51*).

Individual translineage plasticity reprograms have been linked to both RFS and OS (*8*). Here, using only a limited number of genes, we designed a simple specific translineage plasticity gene signature that could be suitable for prognostic purposes. We validated its significance in a cohort of patients with CRC that has not been used in prior studies exploring the correlation between plasticity and survival. We found that this gene signature correlates inversely with survival, with patients showing the highest translineage plasticity scores having the poorest outcomes. In addition, we observed that high *TCF7* expression correlated positively with OS in patients with high translineage plasticity scores, underscoring the clinical relevance of our findings obtained from the preclinical mouse models. However, we did not find a general correlation between *LEF1* expression and survival, suggesting that other mutations or changes at the chromatin level may affect *LEF1*-related survival in late-stage CRC.

To conclude, we show that canonical Wnt signaling represses both progressive intra- and translineage plasticity via maintaining the homeostatic lineage commitment of Lgr5^+^ cells. However, as tumor stem cells in polyps and adenomas show high expression of canonical Wnt target genes and translineage plasticity markers, the role of canonical Wnt signaling in controlling plasticity is more complex than so far described.

## MATERIALS AND METHODS

### Animal experiments

All in vivo experiments were approved by the Regional State Administrative Agency of Finland (ESAVI/22643/2022 and ESAVI/22042/2019). The experiments complied with the guidelines and recommendations of the Federation of European Laboratory Animal Science Association concerning aspects of animal housing, treatment, and euthanasia criteria (maximum 15% weight loss, signs of distress, and blood in feces). The mice were housed in monitoring facilities and had ad libitum access to food and water. Mouse genotypes were checked from ear samples twice before the experiment and once from a tail sample collected after euthanization. The following mouse lines with C57BL/6 background (RRID:MGI:5656552) were used: *Tcf7^GFP^* flox (*52*) (the Jackson Laboratory, stock no. 030909, RRID:IMSR_JAX:030909), *Lef1^fl/fl^* (*53*), *Lgr5-EGFP-IRES-Cre^ERT^* (*17*), *Apc^fl/fl^* (*54*), CAG-FLPe (*55*), Rosa26^LSL-TdTomato^ (*56*) (the Jackson Laboratory, stock no. 021875, RRID:IMSR_JAX:021875), *Apc^Min/+^* (*57*) (the Jackson Laboratory, stock no. 002020), *Kras^LSL-G12D/+^* (*58*), and *Villin-Cre^ERT2^* (*59*) (the Jackson Laboratory, stock no. 020282). All experiments were performed thrice with independent cohorts of approximately equal numbers of male and female mice. 

To induce Cre^ERT2^-mediated deletion of LoxP-targeted genes, 2 mg of tamoxifen (#T5648, Sigma-Aldrich) dissolved in corn oil was administered via intragastric injection for two to three consecutive days. Mice received 0.1 mg of EdU (Invitrogen, A10044) dissolved in 0.9% saline by intraperitoneal injection ~4 hours before euthanasia.

### DNA extraction and polymerase chain reaction

DNA was extracted from mouse ear, tail, and intestinal tissue samples by adding 50 μl of 50 mM sodium hydroxide (S/4920/60, Fisher Chemical) to the tissue samples and incubating at 95°C for 10 min. Polymerase chain reaction (PCR) was performed using PCR KAPA2G Fast HotStart Genotyping mix (KK562107961316001, Kapa Biosystems) and an Applied Biosystems 2720 Thermal cycler with the following program: 3 min at 95°C, 35 cycles of 15 s at 95°C, 15 s at 52°/60°C and 15 to 60 s at 72°C, followed by 3 min at 72°C, and ∞ at 4°C. When using the *Prox1-Cre^ERT2^* primers, a touchdown PCR protocol was used: initial denaturation for 2 min at 95°C, 10 cycles of 15 s at 95°C, 15 s at 65.0 Δ–0.5°C, and 15 s at 68°C (touchdown), followed by 28 cycles of 15 s at 95°C, 15 s at 60°C, and 15 to 60 s at 72°C, ending in final extension step for 3 min at 72°C and ∞ at 10°C. The genotyping primers used are listed in table S5. The PCR fragments were electrophoresed on a 3% agarose gel (BIO-41025, Meridian Bioscience) at 300 V for 30 to 45 min. The amplicon length was estimated by comparison with the GeneRuler ready-to-use 100–base pair (bp) DNA Ladder (SM0243, Thermo Fisher Scientific).

### Sorting of tumor cells for scRNA-seq

Sorting of Epcam^+^ LApc and LApcLef1 tumor cells for previously published scRNA-seq analysis was performed 29 (LApc) or 21 (LApcLef1) days after the first dose of tamoxifen (table S2A) (*28*). In the LApc versus LApcT survival experiments, the first LApcT mice had to be euthanized because of tumor-related well-being issues on day 20 and the last ones on day 30, while the corresponding days for the LApc mice were days 38 and 115. This necessitated the termination and sampling of the LApcT mice analysis on day 19, which was too early for the LApc mice that had a lower tumor burden. To compare mice with a similar tumor burden, we chose days 19 and 68 for the euthanization of the LApcT and LApc mice, respectively. Considering that this might influence the tumor phenotype, we isolated tumor cells from the small intestines of LApcT and LApc mice 19 and 29 days after tamoxifen administration, respectively, and fluorescence-activated cell sorting (FACS)–sorted tdTomato^+^ cells (table S2B).

Small intestines of LApc and LApcT mice were cut open, washed with phosphate-buffered saline (PBS), and incubated in 10 mM EDTA in a shaker at 37°C for 10 min. The tissues were washed twice with PBS, and the cells were scraped off with a glass coverslip. The cells were then centrifuged at 1500 rpm for 5 min, followed by incubation with a digestion mix composed of Hanks’ balanced salt solution (HBSS) with Mg^2+^ and Ca^2+^ (#14025-050, Gibco) and supplemented with 1 mg/ml of collagenase type I (#17100-017, Gibco), collagenase type IV (1 mg/ml) (#17104-019, Gibco), 4 mg/ml of Dispase II (#04942078001, Sigma-Aldrich), and 1 μl/ml of deoxyribonuclease 1 (#EN0521, Thermo Fisher Scientific) for 30 to 40 min at 37°C. After this, Dulbecco’s modified Eagle’s medium (DMEM) was added, and the cell suspension was filtered through a 70-μm and twice through a 40-μm filter and centrifuged for 10 min at 1500 rpm. TdTomato^+^ cells from LApc and LApcT mice were sorted using BD Influx (BD Biosciences) for scRNA-seq and gene expression validation. A total of 10 000 sorted cells were resuspended in HBSS containing 0.04% bovine serum albumin and subjected to Chromium X (RRID:SCR_024537).

### scRNA-seq analysis

#### LApc, LApcT, and LApcLef1

cDNA sample libraries were generated after analysis using Chromium X and sequenced on an Illumina NovaSeq 6000 sequencer (RRID:SCR_016387) at the Single Cell Analytics Unit at the Institute for Molecular Medicine Finland (FIMM). A Cell Ranger (version 6.1.2, 7.0.0, or 7.0.1; RRID:SCR_017344) was used to align the reads, quantify gene expression, generate custom references, and call the cells. The reads were aligned to the mouse reference genome, mm10. Data analysis was performed using RStudio (version 4.2.2, RRID:SCR_000432) following Seurat version 4 and 5 instructions (RRID:SCR007322) (*60*, *61*). To ensure high-quality data for downstream analysis, we applied standardized preprocessing and data-driven quality control criteria. For in-house samples, cells were excluded if they had mitochondrial gene content > 15%, ribosomal RNA content < 1%, total unique molecular identifier (UMI) counts outside the range of 3000 to 85,000 or detected gene counts outside the range of 1000 to 8500. WT samples obtained from Gene Expression Omnibus (GEO; GSE169197) (*29*) cells were excluded if they had a mitochondrial gene content > 10%, total UMI counts > 80,000, or detected gene counts outside the range of 800 to 6500. Genes representing technical biases and ribosomal RNA contamination, including *Malat1*, *Gm26917*, *Gm42418*, and *AY36118*, were excluded from further analyses. Individual samples were normalized using the SCTransform function in Seurat using the glmGamPoi method, which accounts for sequencing depth and other technical variations (RRID:SCR022146) (*62*). Batch effects across samples were corrected using the reciprocal principal components analysis (RPCA) integration method. PCA was performed on the integrated dataset, and the first 30 principal components identified as significant based on the inspection of the ElbowPlot and DimHeatmap visualizations were selected for downstream analysis. Cell clustering was performed using FindNeighbors and FindClusters functions (resolution = 1.6). Genes used for cluster identification are listed in table S3. The ScCustomize R package (RRID:SCR_024675) (*63*) was used to visualize scRNA-seq data.

In the scRNA-seq experiments, two different FACS-based strategies were used to isolate tumor cells. In the *Lef1* deletion experiments [scRNA-seq data previously published (*28*)], dissociated cells were sorted using EpCAM, whereas in the *Tcf7* deletion experiments, sorting was performed on the basis of tdTomato expression. In addition, because the *Lef1* deletion experiment was conducted substantially earlier than the *Tcf7* deletion experiments and because it used a different 10x Genomics reagent chemistry (v2 versus v3), the LApc versus LApcT and LApc versus LApcLef1 analyses were performed separately. However, results from joined analysis with WT, LApc, LApcT, and LApcLef1 samples confirming the main findings of the individual analyses are presented in fig. S10 and in table S6. Total number of all analyzed cells in WT-LApc-LApcT analysis: WT: 12,550, LApc: 15,435, and LApcT: 10,609; and in WT-LApc-LApctLef1 analysis: WT: 10,949, LApc: 10,697, and LApcLef1:16,281. Number of tumor-specific cells in LApc-LApcT analysis: LApc: 855 and LApcT: 3857; and LApc-LApcLef1 analysis: LApc: 1466 and LApcLef1; 9115. Raw and processed scRNA-seq data produced in this study were deposited in GEO database under accession number GSE288156.

#### Healthy tissue, FAP polyp, and carcinoma samples

Healthy tissue samples, FAP polyps, and carcinoma samples from GSE201348 dataset (*27*) were integrated to dataset consisting large intestinal carcinoma samples from patients with average-onset CRC (age at diagnosis ≥ 50 years) (*8*). Cell numbers: healthy: 8303; polyp: 30,105; carcinoma: 3825. Data processing and analysis were performed using Seurat v5 (RRID:SCR007322). Merged objects were normalized using SCTransform, followed by dimensionality reduction with RunPCA. Sample integration was carried out using IntegrateLayers with the RPCA method. Cell clustering was performed using FindNeighbors and FindClusters, and visualization was generated using RunUMAP, using the first 35 principal components. For downstream analyses, epithelial cells (EPCAM > 0) were subsetted from stromal cells. Iterative clustering and subsetting were performed until only epithelial cells were retained in the Seurat object. Epithelial cluster annotation was performed using GSEA with previously published gene signatures defining healthy intestinal epithelial cell types (*8*). Cluster identities were assigned on the basis of significantly enriched signatures, and only annotations with an adjusted *P* < 0.05 were retained.

### TissueEnrich analysis

The TissueEnrich R package (*64*) was used to characterize the cells in tumor-specific clusters. First, we searched for DEGs in the tumor-specific cells between LApcLef1 and LApc samples, from which those with avg_log_2_FC > 1.5 (*n* = 136) or < 1.5 (*n* = 800) were used for the TissueEnrich analysis performed with the default settings. A similar analysis was carried out in LApcT versus LApc tumor–specific cells, but for TissueEnrich, DEGs with avg_log_2_FC > 2.5 (*n* = 164) or < 1.5 (*n* = 1111) were used. The genes used for the analysis are listed in table S3.

### Gene signatures

To analyze whether the WNT score correlates with HiCol or HiSquam signatures (*10*), we quantified the WNT score for each GSE39582 (*30*) patient sample with single-sample GSEA [ssGSEA; gene set variation analysis (GSVA) R package (*65*)] using the MSigDB Wnt/β-catenin signaling Hallmark gene signature. Moreover, *TCF7* and *LEF1* expression correlated with the HiCol and HiSquam signatures. To evaluate this, we divided patient samples based on their WNT score and *TCF7* or *LEF1* expression into three groups: Q3, Q1-Q3, and Q1. We plotted the HiCol and HiSquam signatures against these groups.

### Analysis of tumor cell composition

Differences in cellular composition between LApc and LApcT samples were analyzed using the Bayesian framework scCODA (version 0.1.9) (*66*). The Tuft cell cluster (automatically selected by the tool) and Goblet cell cluster were used as reference cell types. This approach yields consistent results, demonstrating the robustness of our findings. A false discovery rate (FDR) threshold of 0.05 was applied to distinguish credible effects from noncredible effects, in accordance with the package’s default settings.

### Generation of plasticity gene signatures and their correlation with survival in patients with CRC

1) Gene selection and filtering: We began by importing a predefined list of candidate genes containing noncanonical (*8*) and oncofetal (*9*) markers (table S3). These genes were then used to subset the full gene expression matrix of GSE39582 (*30*), retaining only rows corresponding to the selected genes. To ensure biological relevance and reduce noise, we filtered out genes with low variability in expression across the samples. Specifically, we computed the SD of expression for each gene and retained only those with SD > 0.5, indicating sufficient intersample variability.

2) Univariate Cox proportional hazard analysis: The filtered gene set was subjected to univariate Cox proportional hazard regression analysis to evaluate the association between individual gene expression and RFS or OS. Each gene was independently tested using the Cox model, and the results were summarized to extract *P* values, hazard ratios, and confidence intervals. This step enables the identification of genes with potential prognostic significance.

3) Regularized Cox regression via Lasso: Given the high dimensionality of gene expression data and the risk of overfitting in multivariate models, we applied LASSO penalized Cox regression using the glmnet package (*67*). This method simultaneously performs variable selection and regularization, identifying the most predictive subset of genes. Cross-validation was used to determine the optimal penalty parameter (lambda.min), and genes with nonzero coefficients at this optimal lambda were considered significant.

4) Risk score calculation and survival stratification. Using the Lasso-derived coefficients and gene expression data, we computed a risk score for each patient as a linear combination of the selected gene expressions weighted by their model coefficients. Patients with CRC (*n* = 566) were stratified into quartiles based on their risk scores. Risk scores were first converted into numeric values and then divided into four groups (Q1 to Q4) using the 25th, 50th, and 75th percentiles as cutoff values. Quartile 1 (Q1) represented the lowest-risk patients, whereas quartile 4 (Q4) included those with the highest predicted risk. Kaplan-Meier survival analysis was performed across the four quartile groups.

### Joint stratification of *TCF7/LEF1* expression and plasticity scores for survival analysis

On the basis of the plasticity RFS and OS gene signatures, we performed ssGSEA and calculated signature scores for each patient using the ssgseaParam method. The resulting enrichment scores were normalized using the GSVA algorithm to obtain per-sample scores. For each signature, the patients were stratified into four groups (very low, low, moderate, high) based on the quartile distribution of their ssGSEA scores. Quartile cutoff values were computed individually for each signature. To explore the interactive effects of gene-level expression and plasticity scores on patient survival, we performed combined stratification analysis. We assessed *TCF7* and *LEF1* across samples from the expression matrix. Expression values were categorized into quartiles (Q1 to Q4) to define *TCF7* and *LEF1* activity levels. Each sample was then assigned to a combined group representing both its *TCF7* or *LEF1* expression and RFS or OS plasticity signature score quartiles (e.g., Q1_Lowest [gene expression]_Q4_Highest [plasticity score]). We focused on four biologically informative combinations: (i) Q1_Q1: both gene expression and score were low, (ii) Q4_ Q4: both were high, (iii) Q1_ Q4: low gene expression and high pathway activity, and (iv) Q4_ Q1: high gene expression and low pathway activity. These combinations allowed us to investigate additive or antagonistic relationships between gene expression and plasticity score levels in relation to survival outcomes analyzed by Kaplan-Meier survival analysis (*68*, *69*).

### ATAC-seq

ATAC-seq was performed for tdTomato^+^ FACS-sorted tumor cells (table S2C) from four LApc and four LApcLef1 mice following the Omni-ATAC-seq protocol (*70*). Transposition was performed for 50,000 nuclei per sample in triplicates (150,000 nuclei in total) to induce library complexity. DNA fragments for each sample were pooled after transposition and purified using a Zymo DNA Clean and Concentrator-5 kit (catalog no. D4014, Nordic BioSite). Libraries were amplified by PCR and quantified, and the quality was measured by quantitative PCR (qPCR; catalog. no. KK4824, KAPA Library Quantification Kit, Illumina) and TapeStation 2200 (Agilent Technologies). ATAC-seq libraries were sent for 100-bp paired-end Illumina sequencing, aiming for 80 million paired-end reads.

Cutadapt version 1.18 (RRID:SCR_011841) in Trim Galore version 0.6.5 (RRID:SCR_011847), with default parameters, was used for quality and adaptor trimming. Trimmed reads were aligned to the mm10 reference genome using Bowtie 2 version 2.3.5 (RRID:SCR_005476). Reads with a mapping quality below 20 were filtered out, mitochondrial reads were counted, and PCR duplicates were removed using Samtools version 1.9 (RRID:SCR_002105). MACS2 version 2.1.4 was used for peak calling. We created fixed-width peaks ranging 250 bp from the peak summit called with MACS2 callpeak command with parameters “--shiff 75 --extsize 150 --nomodel --call-summits --nolambda --keep-dup all -p 0.01.”

Differential accessibility between groups was calculated using DESeq2 version 1.32.0 (RRID:SCR_015687) with default settings and Wald statistics. To obtain the number of Tn5 insertions, the properly paired paired-end read alignments were corrected for the Tn5 offset (“+” stranded +4 bp, “−” stranded −5 bp) (*71*). Tn5 insertions were counted at nonoverlapping peaks, with the peak with the highest peak score kept in the analysis, for all peaks present in at least two samples. Differentially accessible regions (DARs) were defined with a Benjamini–Hochberg (BH)–adjusted *P* < 0.05. The closest gene for each DAR was annotated using HOMER (RRID:SCR_010881) annotatePeaks.pl against the mm10 RefSeq database (RRID:SCR_003496).

Enrichment of TF motifs to DARs was calculated separately for more accessible [log_2_ fold change (FC) > 0] and less accessible (log_2_ FC < 0) regions using simple enrichment analysis (SEA) (*72*) in MEME Suite (*73*) with default settings. Raw and processed ATAC-seq data produced in this study were deposited in GEO database under accession number GSE288156.

### Tissue processing and immunohistochemistry

Fresh tissue samples were either frozen in liquid nitrogen or fixed overnight (o/n) with 4% paraformaldehyde at 4°C. The following day, tumor numbers were counted, and intestines were rolled into Swiss rolls, dehydrated o/n, embedded in paraffin, and cut into 5-μm sections. Tissue sections were deparaffinized and rehydrated in the following steps: 3× 5-min wash in Tissue-Clear (Tissue-Tek, #1466, Sakura), followed by a decreasing alcohol series (2× absolute ethanol, 2× 96% ethanol, 1× 70% ethanol, and 1× 50% ethanol for 3 min each). This was followed by heat-induced antigen retrieval with high-pH target retrieving solution, 30- to 40-min blocking with TNB buffer (0.1 M tris-HCl, 0.15 M NaCl, and 0.5% TSA blocking reagent, NEL700001KT, PerkinElmer) at room temperature (RT), and primary antibody incubation o/n 4°C. For the anti–c-Myc antibody (RRID:AB_731658), a low-pH (6) Na-citrate–based target retrieval buffer was used, and the primary antibody was incubated for 48 hours. The following primary antibodies were used: rabbit anti-Tcf1 (1:100; #2203, Cell Signaling, RRID:AB_2199302), rabbit anti-Tcf1 (1:200; #MA5-14972, Thermo Fisher Scientific), goat anti-Prox1 (1:200; AF2727, R&D Systems, RRID:AB_2170716), rabbit anti-Lyz1 (1:500; Dako A0099, RRID:AB_2341230), goat anti-Msx1 (1:200; AF5045, R&D Systems, RRID:AB_2148804), rabbit anti-Ki67 (1:200; ab16667, Abcam, RRID:AB_302459), rabbit anti-c-Myc (1:200; Abcam, ab32072, RRID:AB_731658), rabbit anti-Anxa1 (1:200; 71-3400, Invitrogen), and mouse anti–β-catenin (1:200; #0030773, BD Biosciences, RRID:AB_305407). For the detection of EdU, the Click-IT EdU Alexa Fluor 488 Imaging Kit (C10337, Invitrogen) was used according to the manufacturer’s instructions. Specific quantification data from each staining are presented in table S4.

The following day, the sections were washed for 3× 5 min with TNT [0.1 M tris-HCl (pH 7.4), 0.15 M NaCl, and 0.05% Tween 20], incubated in TNB–Alexa Fluor 488–, 594–, and 647– (1:500; Invitrogen) conjugated secondary antibody mix for 60 min at RT, washed again with TNT and Milli-Q water, and mounted with 4′,6-diamidino-2-phenylindole (DAPI) containing VECTASHIELD mounting medium (#H-1200, Vector Laboratories, RRID:AB_2336790) to counterstain the nuclei and lastly sealed under cover glass with cytoseal (#8310-4, Thermo Fisher Scientific). The samples were imaged using an epifluorescence microscope (Zeiss Axio Imager, RRID:SCR_018876) equipped with a Hamamatsu Orca Flash 4.0 LT camera. The original images were processed and analyzed using the Fiji software (RRID:SCR_002285).

### Hematoxylin and eosin staining

Tissue sections were deparaffinized and rehydrated as described above, followed by incubation in MQ water for 1 min and hemalum stock (#1092492500, Merck) for 1 to 2 min, washed under running water for 10 min, and incubated in 1% eosin stock solution in methanol for 30 s. Next, the tissue sections on the slides were dehydrated in an increasing alcohol series (10 s in 70% ethanol, 1 min in 96% ethanol, 2 × 3 min in absolute ethanol, and 3 × 5 min in Tissue-Clear). The slides were mounted with Pertex mounting medium (#00811, Histolab) and imaged in bright field using a Zeiss Axio Imager microscope (RRID:SCR_018876).

### RNA isolation, cDNA synthesis, and qPCR

RNA isolation, cDNA synthesis, and qPCR was performed similarly, as described previously (*23*). In short, RNA isolation from the tumor cells was performed using a NucleoSpin RNA II Kit (#740955, Macherey-Nagel), cDNA was synthesized using a High-Capacity cDNA Reverse Transcription Kit (Thermo Fisher Scientific, #4368814), and RNA transcript expression analysis was performed using qPCR (CFX96 Real-Time System) with a DyNAmo HS SYBR Green qPCR Kit (#F410XL, Thermo Fisher Scientific). The primers used for qPCR are listed in table S5.

### Organoid isolation and culture

To induce tumorigenesis, 2 mg of tamoxifen was administered to LApc and LApcT mice on two consecutive days. After 19 (LApcT) or 29 (LApc) days, mice were euthanized, and the small intestines were harvested. Intestinal tissues were thoroughly washed with PBS, cut into small fragments, and incubated in 10 mM EDTA for 15 min at 4°C. Following a single PBS wash, tissues were subjected to a second incubation in 10 mM EDTA for 45 min at 4°C. During EDTA incubations, samples were vigorously vortexed every 10 to 15 min.

After incubation, tissue fragments were further vortexed and allowed to settle, and the supernatant containing released crypts was collected. The supernatant was centrifuged at 1500 rpm for 5 min. Isolated crypts were embedded in Matrigel (#356231, Corning), and after Matrigel polymerization, cultures were overlaid with Advanced DMEM/F12 (#12634-010, Gibco) supplemented with B27 (#17504-044, Gibco), N2 (#17502-048, Gibco), 10 mM Hepes (#BP310-1, Thermo Fisher Scientific), 1 mM *N*-acetylcysteine (#A-9165, Sigma-Aldrich), Penicillin G (100 IU/ml)/streptomycin sulfate (100 μg/ml) (ECB001D, Euroclone), and 2 mM l-glutamine (ECB3000D, Euroclone). Experiments were conducted once organoid formation and growth had stabilized.

### Statistical analysis

The number of technical and biological replicates is indicated in the figure legends. Data are presented as mean ± SD unless otherwise indicated in the figure legend. GraphPad Prism (version 9, RRID:SCR_002798) and RStudio (version 4.2.2, RRID:SCR_000432) were used for statistical analyses. Statistical analysis comparing groups was performed using Student’s unpaired *t* test, Mann-Whitney test, or two-way analysis of variance (ANOVA). Selected statistical analyses are indicated in the figure legends. Statistical significance is indicated by *P* values (*P < 0.05, **P < 0.01, ***P < 0.005, and ****P < 0.001). RStudio (version 4.2.2) was used for statistical analyses.
