# Supplementary material for: Progressive intestinal tumor cell plasticity, Myc activation, and loss of Lgr5+ tumor stem cell lineage commitment upon Wnt depletion
Source: Sci Adv. 2026 Jul 10;12(28):eaeb8564. doi: 10.1126/sciadv.aeb8564 (PMC13353423; doi:10.1126/sciadv.aeb8564)
Supplement: Supplementary file 1 — Figs. S1 to S10 Legends for tables S1 to S6 [file sciadv.aeb8564_sm.pdf]

Science Advances
AAAS

Supplementary Materials for

Progressive intestinal tumor cell plasticity, Myc activation, and loss of Lgr5$^+$ tumor stem cell lineage commitment upon Wnt depletion

Marika Lassila *et al.*

Corresponding author: Pauliina Kallio, pauliina.kallio@helsinki.fi; Kari Alitalo, kari.alitalo@helsinki.fi

Sci. Adv. **12**, eaeb8564 (2026)
DOI: 10.1126/sciadv.aeb8564

The PDF file includes:

Figs. S1 to S10
Legends for tables S1 to S6

Other Supplementary Material for this manuscript includes the following:

Tables S1 to S6

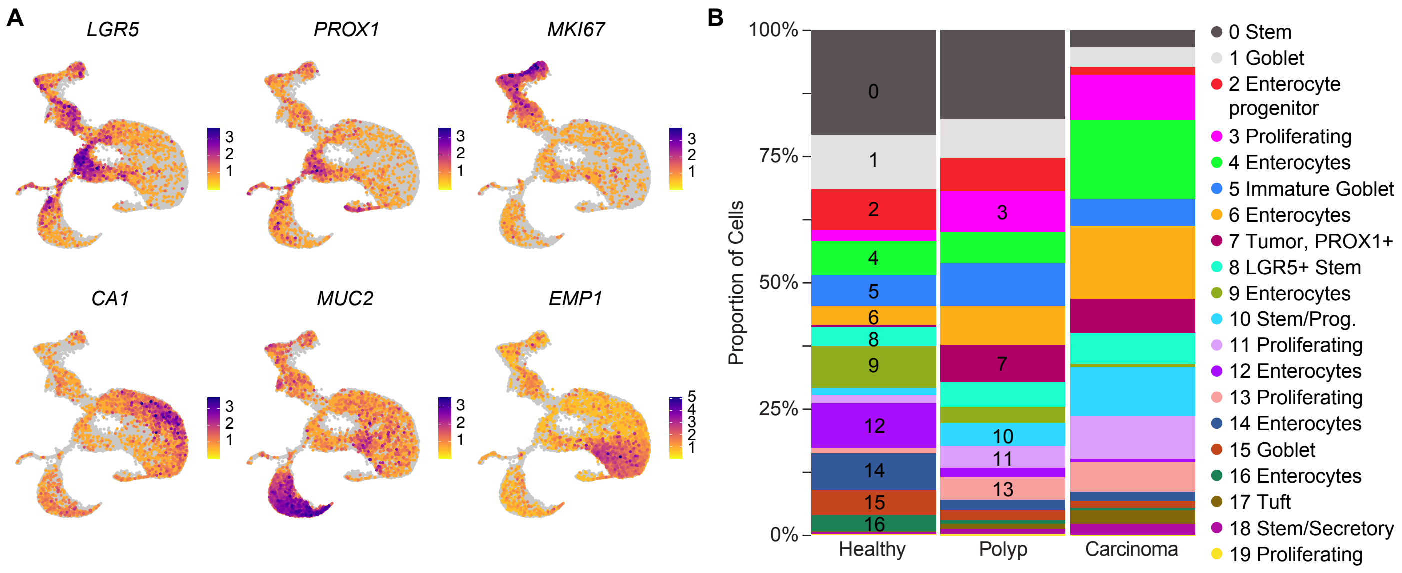
A
LGR5
PROX1
MKI67
CA1
MUC2
EMP1
B
100%
75%
50%
25%
0%
Proportion of Cells
Healthy
Polyp
Carcinoma
0 Stem
1 Goblet
2 Enterocyte progenitor
3 Proliferating
4 Enterocytes
5 Immature Goblet
6 Enterocytes
7 Tumor, PROX1+
8 LGR5+ Stem
9 Enterocytes
10 Stem/Prog.
11 Proliferating
12 Enterocytes
13 Proliferating
14 Enterocytes
15 Goblet
16 Enterocytes
17 Tuft
18 Stem/Secretory
19 Proliferating

Supp. Figure S1. Cell clustering in patient samples. A, Feature plots showing the expression of *LGR5*, *PROX1*, *MKI67*, *CA1*, *MUC2*, and *EMP1* in the indicated samples. The color scale indicates gene expression levels. **B,** Proportion of cell clusters in healthy, polyp and carcinoma samples.

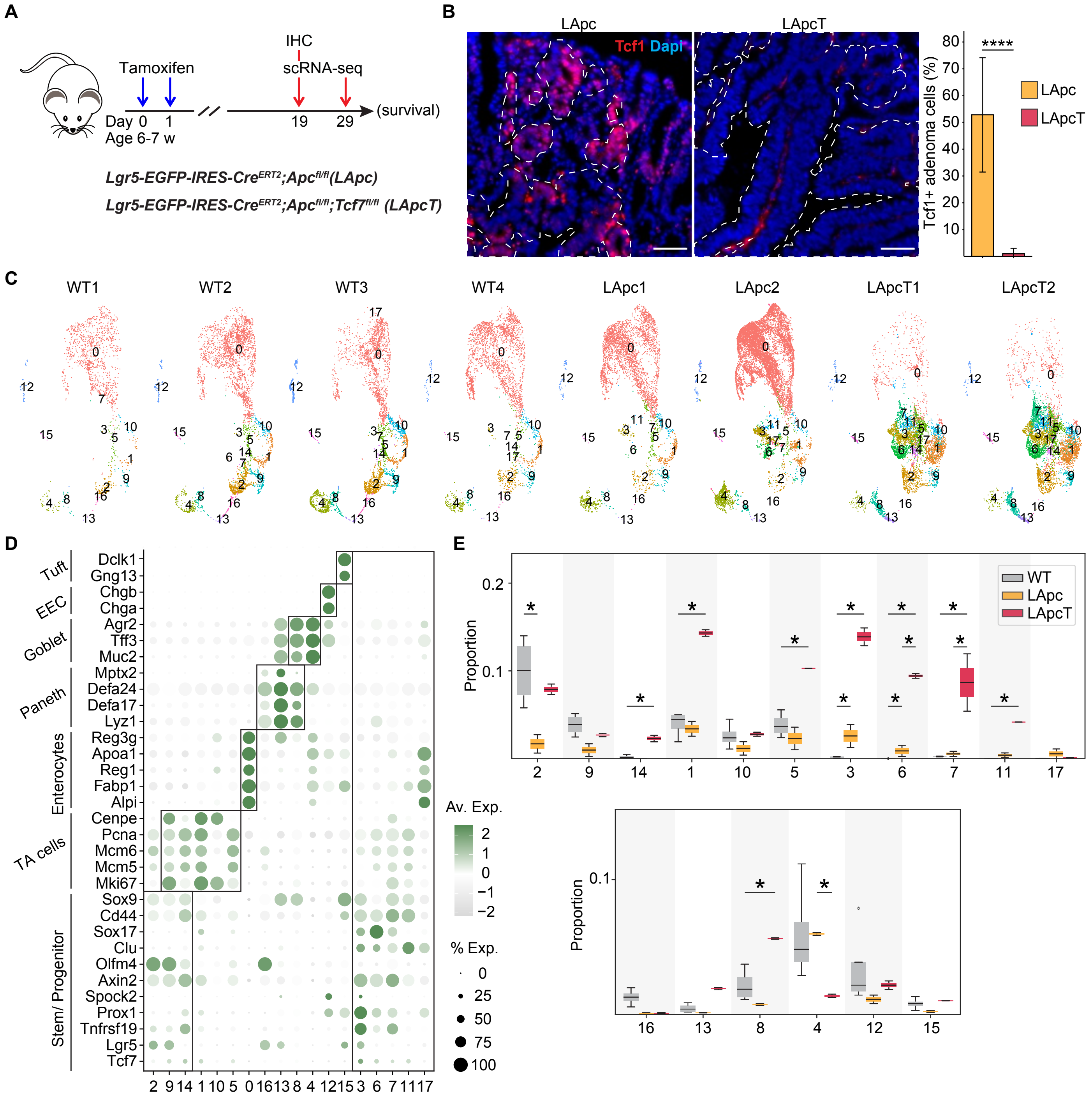
A
Tamoxifen
IHC
scRNA-seq
Day 0 1 19 29 (survival)
Age 6-7 w
Lgr5-EGFP-IRES-CreERT2;Apcfl/fl(LApc)
Lgr5-EGFP-IRES-CreERT2;Apcfl/fl;Tcf7fl/fl (LApcT)
B
LApc
LApcT
Tcf1 Dapi
Tcf1+ adenoma cells (%)
****
LApc
LApcT
C
WT1
WT2
WT3
WT4
LApc1
LApc2
LApcT1
LApcT2
D
Tuft
EEC
Goblet
Paneth
Enterocytes
TA cells
Stem/ Progenitor
Dclk1
Gng13
Chgb
Chga
Agr2
Tff3
Muc2
Mptx2
Defa24
Defa17
Lyz1
Reg3g
Apoa1
Reg1
Fabp1
Alpi
Cenpe
Pcna
Mcm6
Mcm5
Mki67
Sox9
Cd44
Sox17
Clu
Olfm4
Axin2
Spock2
Prox1
Tnfrsf19
Lgr5
Tcf7
Av. Exp.
% Exp.
E
Proportion
WT
LApc
LApcT

Supp. Figure S2. ***Tcf7*** **deletion increases the proportion of proliferating tumor cells. A,** Schematic of the experiment. **B,** Immunofluorescence staining and quantification of Tcf1+ tumor cells in the LApc and LApcT samples 19 days after tamoxifen administration. Scale bars: 50 μm. The dashed lines indicate the nuclear β-catenin+ tumor areas. The bar plots show average +/- SD. Number of mice: LApc: 12, LApcT: 12. Number of quantified tumors: LApc: 77, LApcT: 113. ****p-value 2.3 $*10^{-63}$, using student's t-test. **C,** Dim plots showing clustering of individual WT, LApc, and LApcT samples. Three of the WT samples (WT1-3) were uploaded from GSE169197. Annotation of the cells: 0: enterocyte, 1: TA 1, 2: Stem cells 3: Tumor stem/progenitor 4: Goblet 5: TA 2, 6: Tumor 1, 7: Tumor 2, 8: Goblet/Paneth, 9: TA 3, 10: TA 4, 11: Tumor 3, 12: EEC, 13: Paneth 1, 14: TA 5, 15: Tuft, 16: Paneth 2, 17: Tumor 4. **D,** Dot plot showing gene expressions of some of the genes used to identify the cell clusters. The size of the dot presents proportion of cells expressing the indicated transcript and the color scale indicates gene expression levels. Black rectangle on the right side of the dot plot indicates the tumor-specific clusters. **E,** Proportion of indicated cell clusters in WT (grey), LApc (orange), and LApcT (red) samples. Credible and significant are denoted by * (FDR < 0.05).

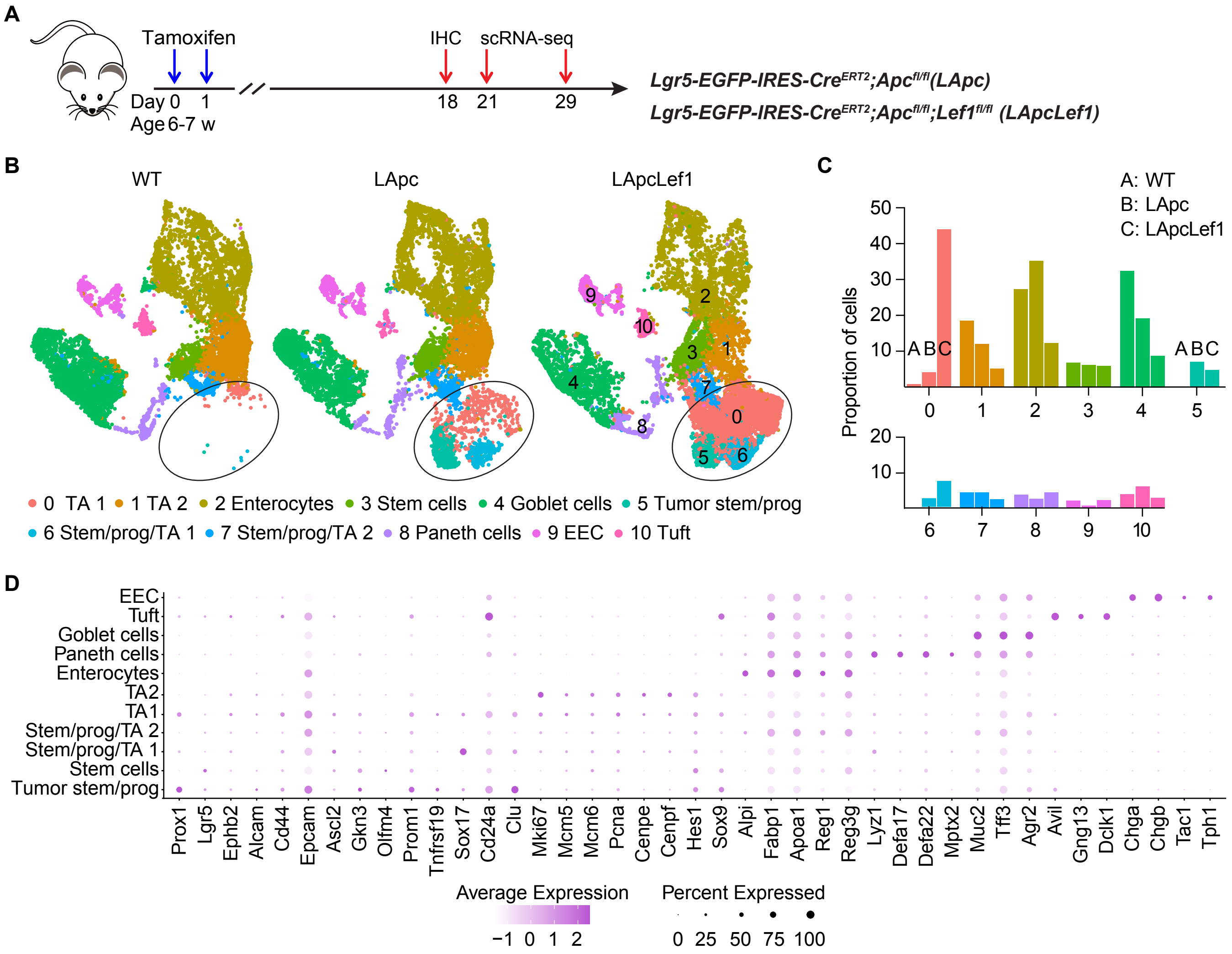

A
Tamoxifen
IHC
scRNA-seq
Day 0 1
Age 6-7 w
18 21 29
Lgr5-EGFP-IRES-CreERT2;Apcfl/fl(LApc)
Lgr5-EGFP-IRES-CreERT2;Apcfl/fl;Lef1fl/fl (LApcLef1)
B
WT
LApc
LApcLef1
0 TA 1
1 TA 2
2 Enterocytes
3 Stem cells
4 Goblet cells
5 Tumor stem/prog
6 Stem/prog/TA 1
7 Stem/prog/TA 2
8 Paneth cells
9 EEC
10 Tuft
C
A: WT
B: LApc
C: LApcLef1
Proportion of cells
D
EEC
Tuft
Goblet cells
Paneth cells
Enterocytes
TA2
TA1
Stem/prog/TA 2
Stem/prog/TA 1
Stem cells
Tumor stem/prog
Prox1 Lgr5 Ephb2 Alcam Cd44 Epcam Ascl2 Gkn3 Olfm4 Prom1 Tnfrsf19 Sox17 Cd24a Clu Mki67 Mcm5 Mcm6 Pcna Cenpe Cenpf Hes1 Sox9 Alpi Fabp1 Apoa1 Reg1 Reg3g Lyz1 Defa17 Defa22 Mptx2 Muc2 Tff3 Agr2 Avil Gng13 Dclk1 Chga Chgb Tac1 Tph1
Average Expression
−1 0 1 2
Percent Expressed
0 25 50 75 100

Supp. Figure S3. *Lef1* deletion in *Apc* mutant tumor cells increases proportion of proliferating cells and decreases proportion of enterocyte-like cells. A, Schematic of the experiment. **B,** Dim plots showing the annotated cell clusters in the indicated samples. **C**, Quantification of the proportions of the cell clusters in the WT (A), LApc (B), and LApcLef1(C) samples. Note, that the color of the bars indicates the cell cluster presented in (**B**). **D**, Dot plot showing gene expressions of some of the genes used to identify the cell clusters. The size of the dot presents proportion of cells expressing the indicated transcript and the color scale indicates gene expression levels.

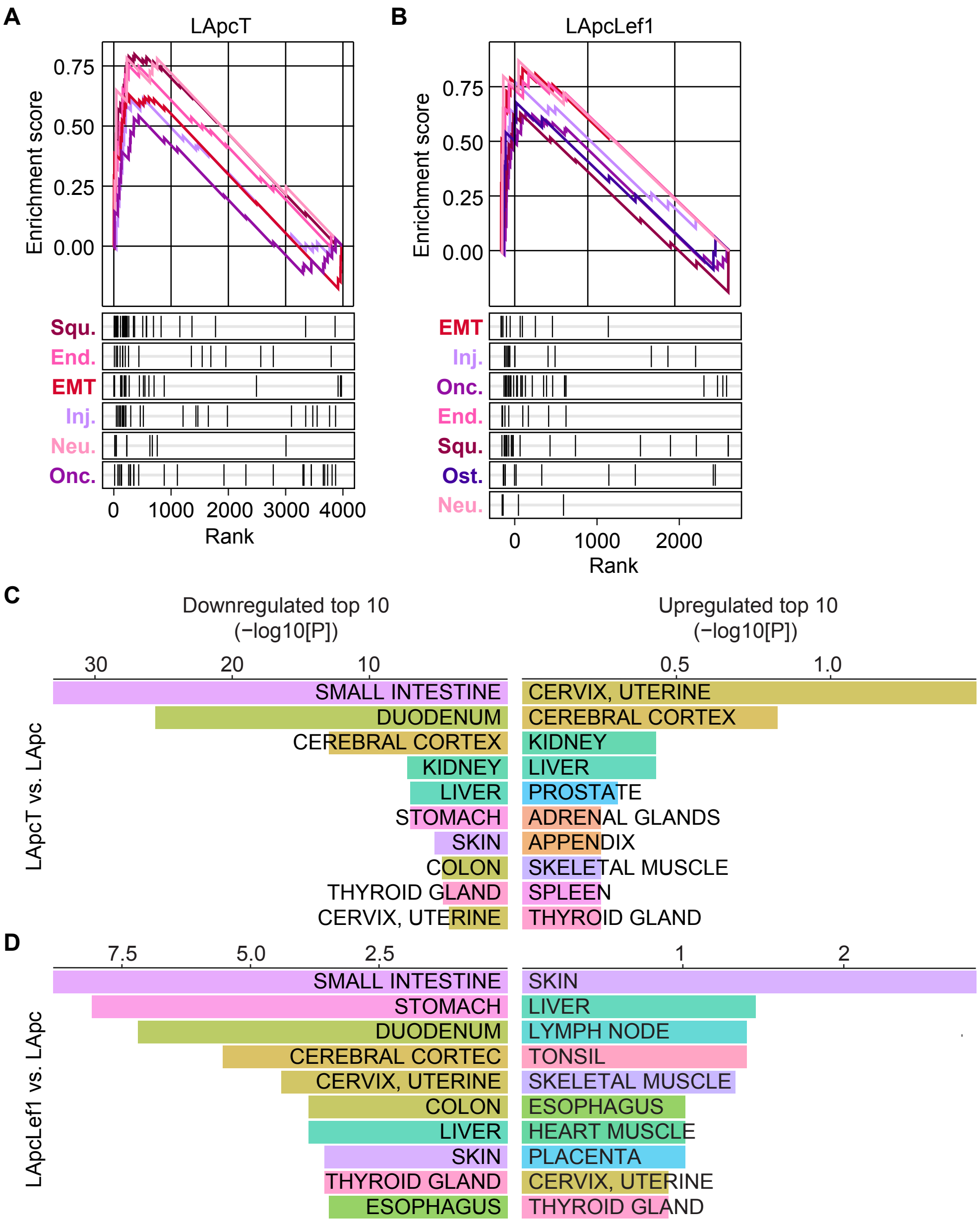

A
LApcT
Enrichment score
0.75
0.50
0.25
0.00
Squ.
End.
EMT
Inj.
Neu.
Onc.
0 1000 2000 3000 4000
Rank
B
LApcLef1
Enrichment score
0.75
0.50
0.25
0.00
EMT
Inj.
Onc.
End.
Squ.
Ost.
Neu.
0 1000 2000
Rank
C
Downregulated top 10
(−log10[P])
30 20 10
Upregulated top 10
(−log10[P])
0.5 1.0
LApcT vs. LApc
SMALL INTESTINE
DUODENUM
CEREBRAL CORTEX
KIDNEY
LIVER
STOMACH
SKIN
COLON
THYROID GLAND
CERVIX, UTERINE
CERVIX, UTERINE
CEREBRAL CORTEX
KIDNEY
LIVER
PROSTATE
ADRENAL GLANDS
APPENDIX
SKELETAL MUSCLE
SPLEEN
THYROID GLAND
D
7.5 5.0 2.5
1 2
LApcLef1 vs. LApc
SMALL INTESTINE
STOMACH
DUODENUM
CEREBRAL CORTEC
CERVIX, UTERINE
COLON
LIVER
SKIN
THYROID GLAND
ESOPHAGUS
SKIN
LIVER
LYMPH NODE
TONSIL
SKELETAL MUSCLE
ESOPHAGUS
HEART MUSCLE
PLACENTA
CERVIX, UTERINE
THYROID GLAND

Supp. Figure S4. Non-canonical pathways are enriched in *Tcf7* or *Lef1* deleted tumor samples. A, LApcT cluster 1 enrichment scores for squamous [Squ.], endoderm [End.], EMT, injury repair [Inj.], neuroendocrine [Neu.], and oncofetal [Onc.] signatures. p-adj. < 0.05, specific padj-values and normalized enrichment scores are presented in **Supp. Table S3**. Enrichment scores were quantified using the fgsea R package. For statistical analysis, Benjamini-Hochberg FDR correction was used. **B,** LApcLef1 cluster 1 enrichment scores for EMT, Inj., Onc., End., Squ., osteoblast [Ost.], and Neu. signatures. p-adj. < 0.05, specific padj-values and normalized enrichment scores are presented in **Supp. Table S3**. **C, D**, TissueEnrich analysis of enriched and downregulated gene signatures in the (**C**) LApcT and (**D**), LApcLef1 samples compared to LApc controls. Top 10 results based on -log10(P-value) are presented.

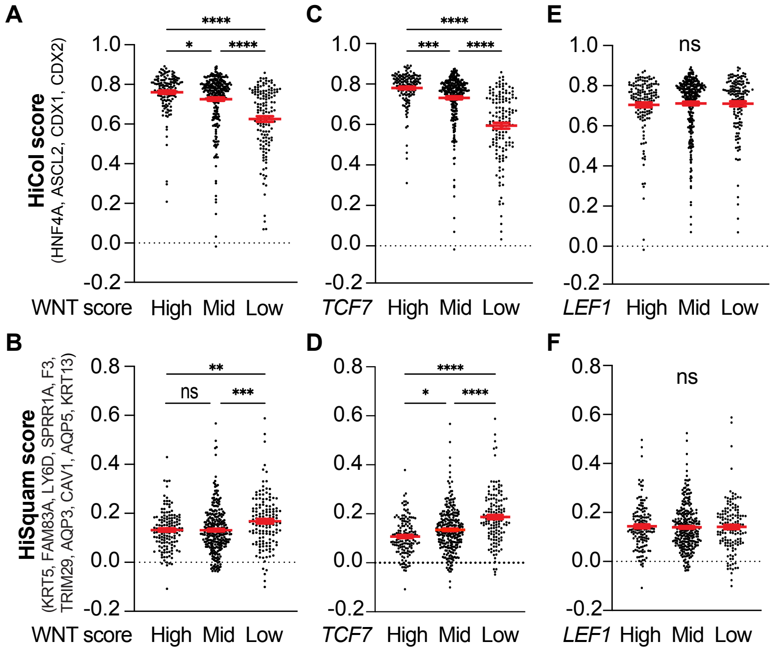

A
B
C
D
E
F
HiCol score
(HNF4A, ASCL2, CDX1, CDX2)
HiSquam score
(KRT5, FAM83A, LY6D, SPRR1A, F3, TRIM29, AQP3, CAV1, AQP5, KRT13)
WNT score
TCF7
LEF1
High
Mid
Low
****
*
***
**
ns
1.0
0.8
0.6
0.4
0.2
0.0
-0.2

Supp. Figure S5. Squamous gene signature correlates negatively with WNT score and *TCF7* expression. A-F, HiCol and HiSquam signatures (10) including the listed genes were quantified for each GSE39583 CRC patient sample by using the ssGSEA GSVA R package. Also, *TCF7* and *LEF1* expression was quantified for each patient sample. The patient samples were assigned into groups based on their WNT score or *TCF7* or *LEF1* expression. Samples in "Low" stand for < 0.25 percentile, and "High" for > 0.75 percentile, and "Mid" for 0.25-0.75 (presented in x-axis). These were plotted against the (**A, C, E**) HiCol and (**B, D, F**) HiSquam signatures. Number of patients in High: 142, Mid: 282, Low: 142. One-way ANOVA and Tuke's multiple comparisons test were used. The p-values are following: (**A**) * 0.0363, **** < 0.0001, (**B**) ** 0.0037, *** 0.0005, (**C)**, *** 0.0008, **** < 0.0001, (**D**) **** < 0.0001. Red line represents the average gene signature expression.

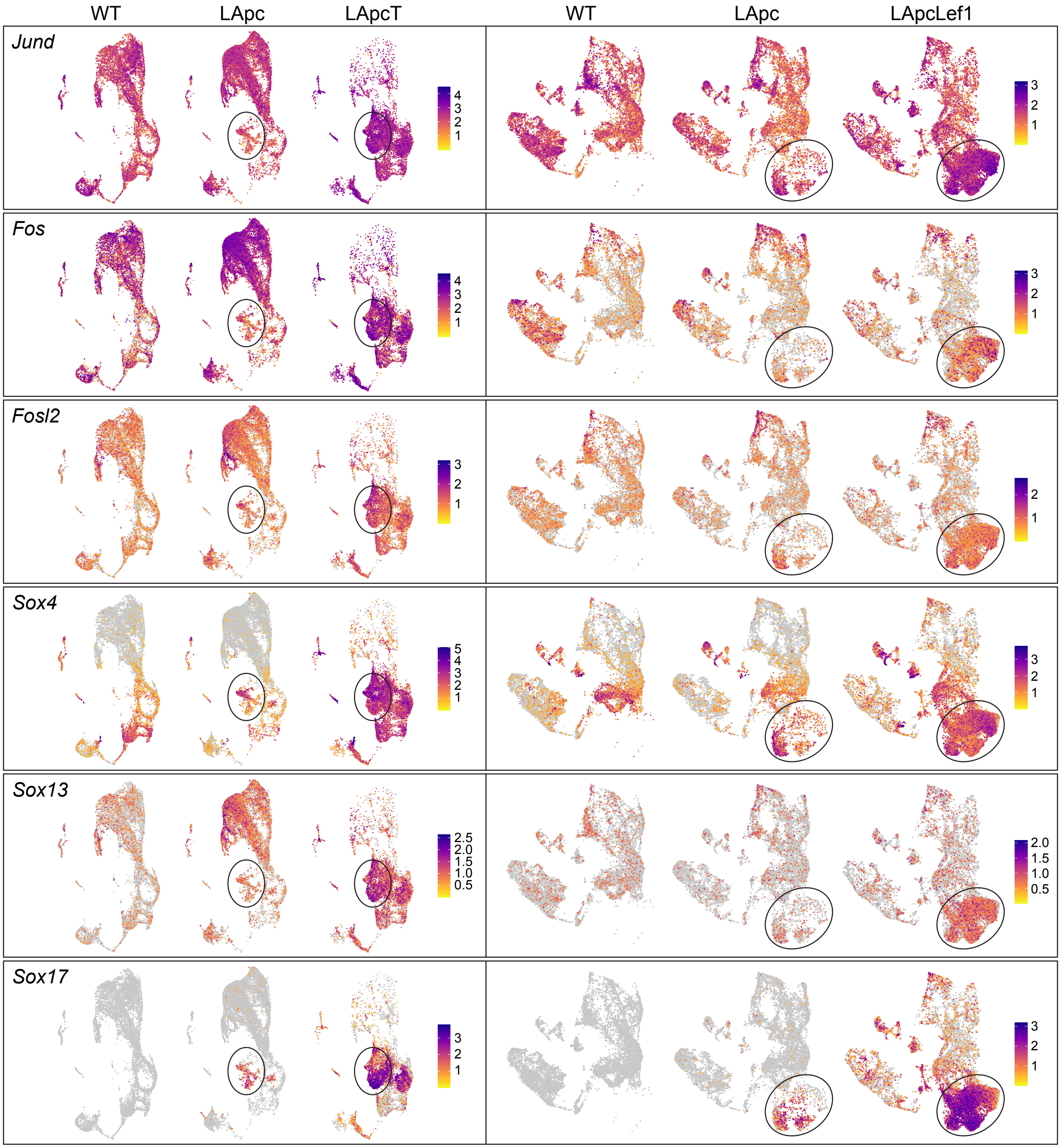
WT
LApc
LApcT
WT
LApc
LApcLef1
Jund
Fos
Fosl2
Sox4
Sox13
Sox17

Supp. Figure S6. Increased expression of AP-1 subunits and Sox family members after *Tcf7* or *Lef1* deletion in *Apc* mutant tumors. Feature plots showing the expression of *Jun*, *Fos*, *Fosb*, *Fosl2*, *Sox4*, *Sox13*, and *Sox17* in the indicated samples. The tumor-specific clusters are encircled. The color scale indicates gene expression levels. Statistical significances were quantified with one-way Anova and Tukey post-hoc test with the Benjamini-Hochberg method and are presented in **Supp. Table S3**.

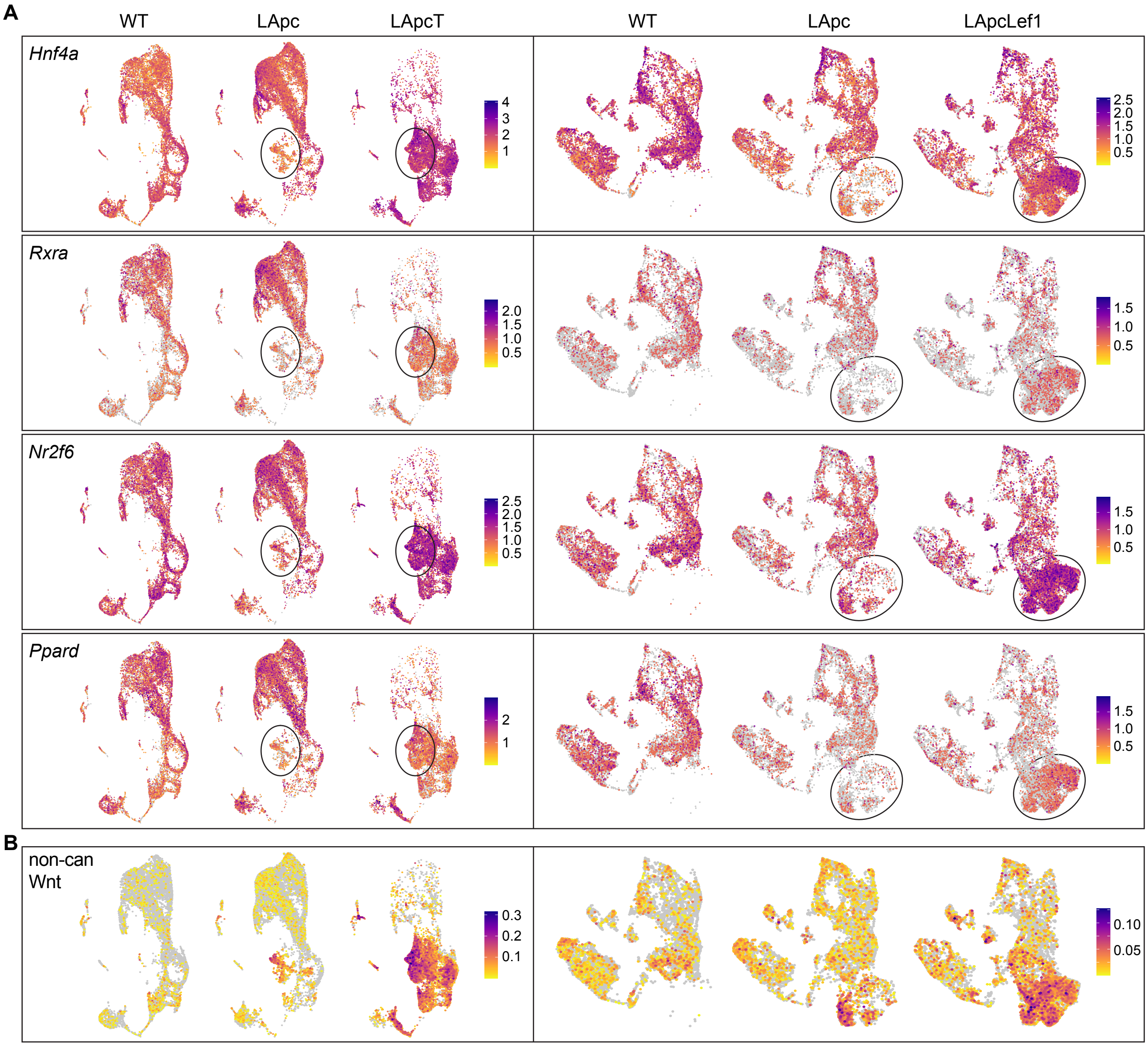
A
WT
LApc
LApcT
WT
LApc
LApcLef1
Hnf4a
4
3
2
1
2.5
2.0
1.5
1.0
0.5
Rxra
2.0
1.5
1.0
0.5
1.5
1.0
0.5
Nr2f6
2.5
2.0
1.5
1.0
0.5
1.5
1.0
0.5
Ppard
2
1
1.5
1.0
0.5
B
non-can
Wnt
0.3
0.2
0.1
0.10
0.05

Supp. Figure S7. Expression of protectors of intestinal epithelial fidelity after *Tcf7* or *Lef1* deletion in *Apc* mutant tumors. A, Feature plots showing the expression of *Hnf4a*, *Rxra*, *Nr2f6*, and *Ppard* in the indicated samples. The tumor-specific clusters are encircled. The color scale indicates gene expression levels. Statistical significances were quantified with one-way Anova and Tukey post-hoc test with the Benjamini-Hochberg method and are presented in **Supp. Table S3**. **B**, Module enrichment score (MES) plots showing expression of the non-canonical Wnt gene signature in indicated samples. The color scale indicates the MES levels.

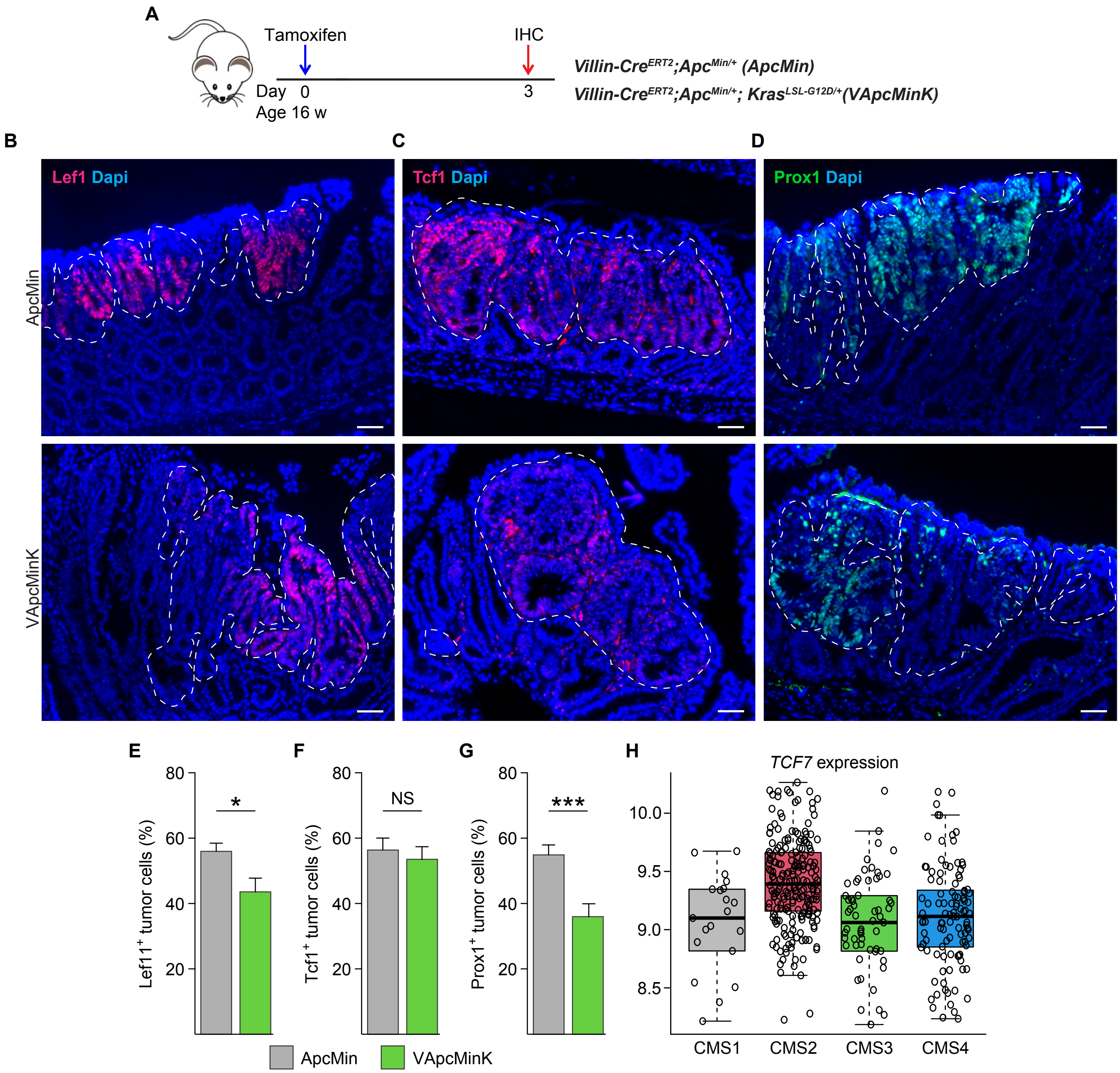
A
Tamoxifen
IHC
Day 0
3
Age 16 w
Villin-CreERT2;ApcMin/+ (ApcMin)
Villin-CreERT2;ApcMin/+; KrasLSL-G12D/+(VApcMinK)
B
C
D
Lef1 Dapi
Tcf1 Dapi
Prox1 Dapi
ApcMin
VApcMinK
E
F
G
H
Lef11+ tumor cells (%)
Tcf1+ tumor cells (%)
Prox1+ tumor cells (%)
*
NS
***
ApcMin
VApcMinK
TCF7 expression
CMS1
CMS2
CMS3
CMS4

Supp. Figure S8. ***Kras*** **mutation decreases Lef1 and Prox1 expression in** ***Apc***$^{\boldsymbol{Min/+}}$ **mice. A,** Schematic of the experiment. **B-D**, Immunofluorescence stainings of (**B**) Lef1+ (red), (**C**) Tcf1+ (red), and (**D**) Prox1+ (green) tumor cells in ApcMin and VApcMinK samples 3 days after tamoxifen administration. Scale bars: 50 μm. The dashed lines indicate the nuclear β-catenin+ tumor areas. **E-G**, Quantifications of the (**E**) Lef1+, (**F**) Tcf1+, and (**G**) Prox1+ ApcMin (grey) and VApcMinK (green) tumor cells. P-values were quantified with unpaired t-test: *0.0491, ***0.0003. Average +/- SD are shown in the bar plots. **H**, *TCF7* expression analyzed in CRC patient-derived bulk-RNA-seq in CMS1-4 subtypes. Each dot presents one patient sample. P-value for CMS2 vs. CMS3: $6.3*10^{-10}$, other p-values are presented in **Supp. Table S3**. Statistical significances were quantified with one-way Anova and Tukey post-hoc test.

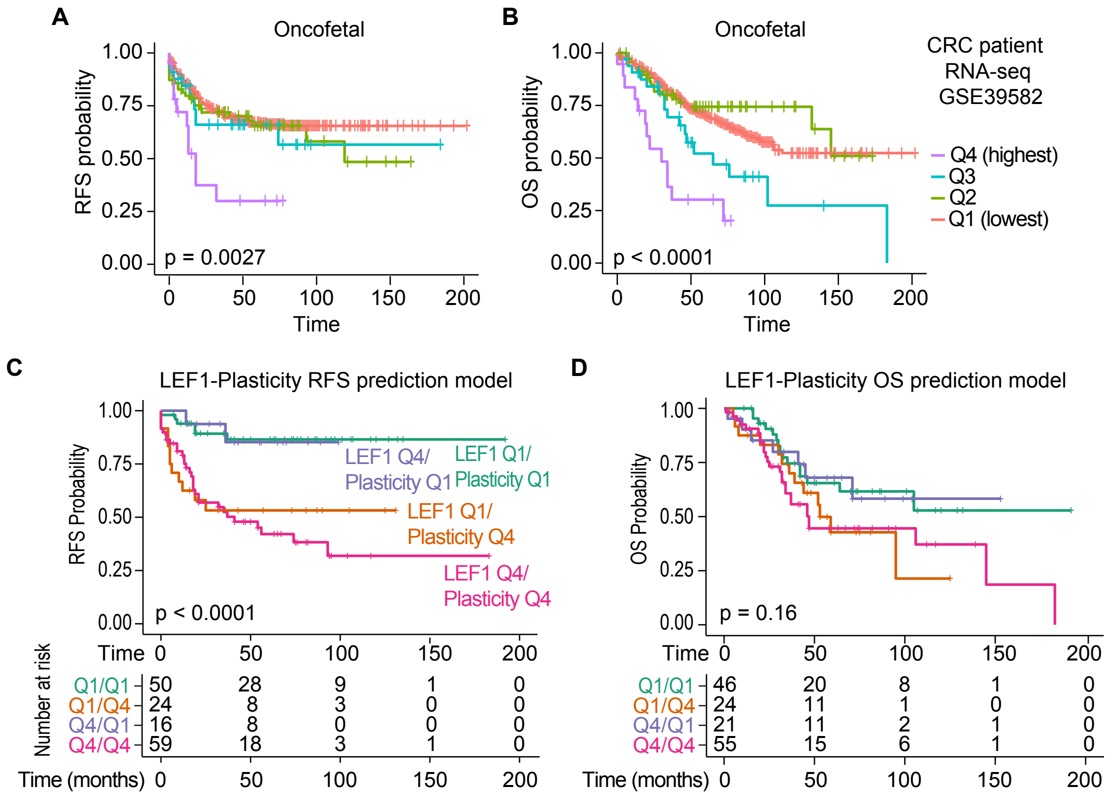

A
Oncofetal
RFS probability
1.00
0.75
0.50
0.25
0.00
p = 0.0027
0
50
100
150
200
Time
B
Oncofetal
OS probability
p < 0.0001
Time
CRC patient
RNA-seq
GSE39582
Q4 (highest)
Q3
Q2
Q1 (lowest)
C
LEF1-Plasticity RFS prediction model
RFS Probability
LEF1 Q4/
Plasticity Q1
LEF1 Q1/
Plasticity Q1
LEF1 Q1/
Plasticity Q4
LEF1 Q4/
Plasticity Q4
p < 0.0001
Number at risk
Time 0 50 100 150 200
Q1/Q1 50 28 9 1 0
Q1/Q4 24 8 3 0 0
Q4/Q1 16 8 0 0 0
Q4/Q4 59 18 3 1 0
Time (months) 0 50 100 150 200
D
LEF1-Plasticity OS prediction model
OS Probability
p = 0.16
Time 0 50 100 150 200
Q1/Q1 46 20 8 1 0
Q1/Q4 24 11 1 0 0
Q4/Q1 21 11 2 1 0
Q4/Q4 55 15 6 1 0
Time (months) 0 50 100 150 200

Supp. Figure S9. High oncofetal score correlates with poor survival in CRC patients. A, B, Oncofetal scores were quantified for each GSE39582 CRC patient sample. Top 10 most expressed genes of the indicated gene signatures among the patient samples were used here to quantify the scores by using ssGSEA and GSVA R packages. (**A**) Recurrence-free survival (RFS) or (**B**) overall survival (OS) probability was quantified for Q1 (< 0.25 percentile, red line), Q2 (green line), Q3 (blue line), and Q4 (> 0.75 percentile, purple line). Probabilities were quantified by using survival (68) and survminer (69) R packages. Statistical significances were quantified by using Cox proportional hazards regression model. **C,D,** Analysis of RFS and OS plasticity scores and *LEF1* expression to survival was performed similarly as in **Figure 8D-F**. **C**,**D,** RFS (**C**) and OS (**D**) probabilities between Q1 (lowest *LEF1*) -Q1 (lowest plasticity score) (green line), Q1-Q4 (orange line), Q4-Q4 (pink line), and Q4-Q1 (purple line). Probabilities were quantified with survival and survminer R packages. Statistical significances were quantified with Cox proportional hazards regression model.

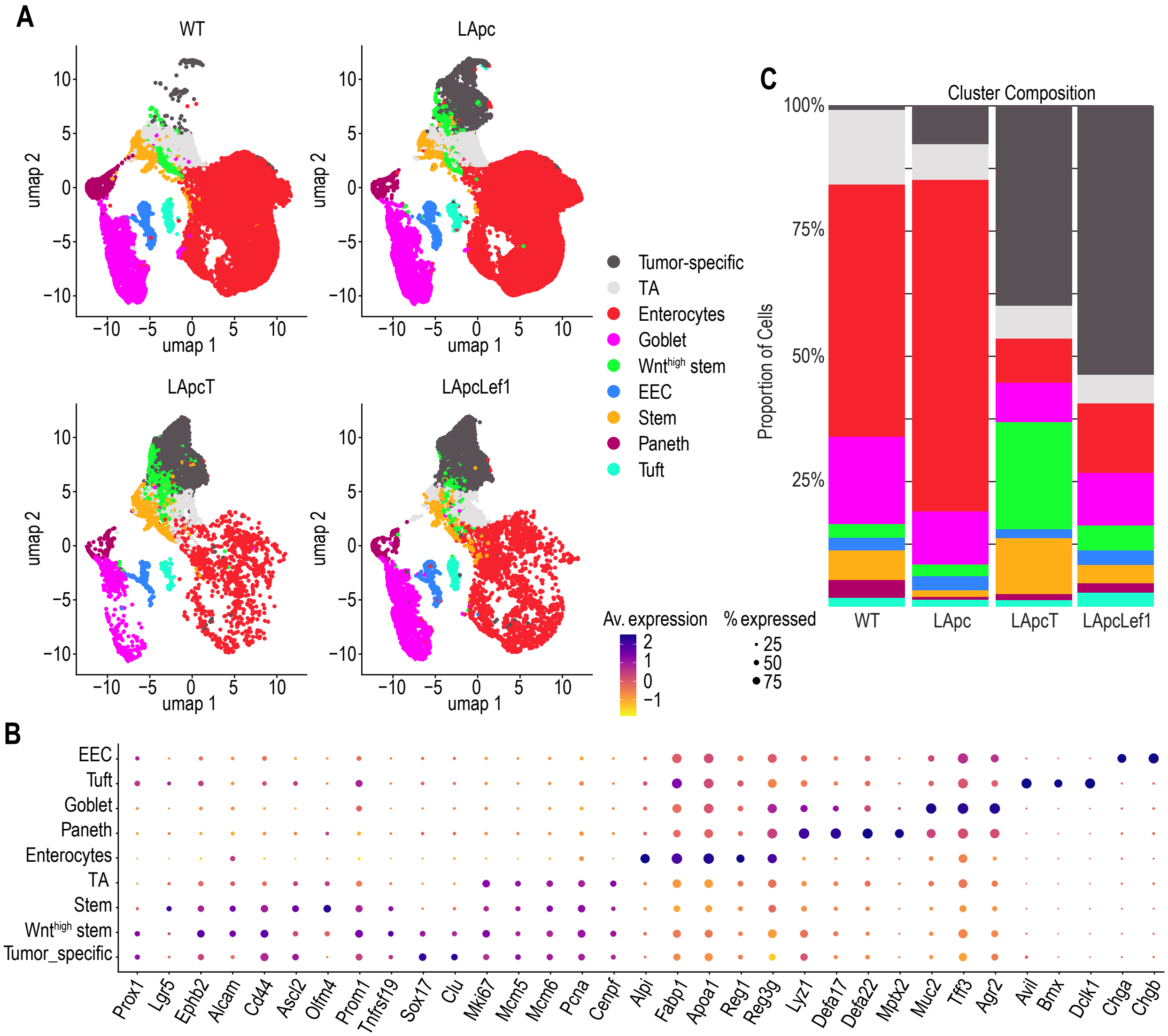

A
WT
LApc
LApcT
LApcLef1
umap 1
umap 2
Tumor-specific
TA
Enterocytes
Goblet
Wnt^high stem
EEC
Stem
Paneth
Tuft
Av. expression
% expressed
25
50
75
C
Cluster Composition
Proportion of Cells
100%
75%
50%
25%
B
EEC
Tuft
Goblet
Paneth
Enterocytes
TA
Stem
Wnt^high stem
Tumor_specific
Prox1
Lgr5
Ephb2
Alcam
Cd44
Ascl2
Olfm4
Prom1
Tnfrsf19
Sox17
Clu
Mki67
Mcm5
Mcm6
Pcna
Cenpf
Alpi
Fabp1
Apoa1
Reg1
Reg3g
Lyz1
Defa17
Defa22
Mptx2
Muc2
Tff3
Agr2
Avil
Bmx
Dclk1
Chga
Chgb

Supp. Figure S10. Failure of Lgr5$^+$ cells to commit to enterocyte-like tumor cell lineage upon *Tcf7* or *Lef1* deletion. A, B, UMAP visualization (**A**) of the epithelial cells in joined analysis with WT, LApc, LApcT, and LApcLef1 samples. Clusters have been annotated with known cluster markers, and expression of some of them is presented in the dot plot (**B**). Total number of analyzed cells: WT, 25,157; LApc, 35,472; LApcT 12,625, and LApcLef1:16,615. **C,** Cluster proportions presented in the indicated samples. Specific proportions are presented in **Supp. Table S6**.

Legends for Supplementary Tables

Lassila Supplementary Table S1: DEGs from healthy, FAP polyp, and CRC sample clusters. Differentially expressed genes (DEGs) between the clusters presented in Fig. 1A.

Lassila Supplementary Table S2: Flow cytometry gating for scRNA-seq and ATAC-seq experiments. Presented are flow cytometry gating strategies for WT, LApc, LApcT, and LApcLef1 samples that were sorted for scRNA-seq or ATAC-seq experiments.

Lassila Supplementary Table S3: Detailed results from WT, LApc, LApcT, LApcLef1 scRNA-seq experiments. Listed are detailed results related to the scRNA-seq analyses.

Lassila Supplementary Table S4: Detailed IF quantification results. Listed are individual values for Prox1, Tcf1, EdU, Myc, Msx1, and Anxa1 immunofluorescence staining quantifications. Each color (blue, green, or lilac) denotes a separate experiment. The total number of mice and tumors analyzed for each staining is indicated, along with the number of mice used in each individual experiment. The results of the t-test comparing LApc and LApcT for each staining are also shown.

Lassila Supplementary Table S5: Genotyping and qPCR primers. Listed are forward and reverse primers used for genotyping or qPCR.

Lassila Supplementary Table S6: Cluster proportions from joined tumor scRNA-seq analysis. Presented are cluster proportions in the indicated cell types in WT, LApc, LApcT, and LApcLef1 samples in the integrated scRNA-seq analysis.
